# Origin and evolutionary history of freshwater Rhodophyta: further insights based on phylogenomic evidence

**DOI:** 10.1038/s41598-017-03235-5

**Published:** 2017-06-07

**Authors:** Fangru Nan, Jia Feng, Junping Lv, Qi Liu, Kunpeng Fang, Chaoyan Gong, Shulian Xie

**Affiliations:** School of Life Science, Shanxi University, Taiyuan, (030006) China

## Abstract

Freshwater representatives of Rhodophyta were sampled and the complete chloroplast and mitochondrial genomes were determined. Characteristics of the chloroplast and mitochondrial genomes were analyzed and phylogenetic relationship of marine and freshwater Rhodophyta were reconstructed based on the organelle genomes. The freshwater member *Compsopogon caeruleus* was determined for the largest chloroplast genome among multicellular Rhodophyta up to now. Expansion and subsequent reduction of both the genome size and GC content were observed in the Rhodophyta except for the freshwater *Compsopogon caeruleus*. It was inferred that the freshwater members of Rhodophyta occurred through diverse origins based on evidence of genome size, GC-content, phylogenomic analysis and divergence time estimation. The freshwater species *Compsopogon caeruleus* and *Hildenbrandia rivularis* originated and evolved independently at the inland water, whereas the *Bangia atropurpurea*, *Batrachospermum arcuatum* and *Thorea hispida* are derived from the marine relatives. The typical freshwater representatives Thoreales and Batrachospermales are probably derived from the marine relative *Palmaria palmata* at approximately 415–484 MYA. The origin and evolutionary history of freshwater Rhodophyta needs to be testified with more organelle genome sequences and wider global sampling.

## Introduction

Rhodophyta is an anciently derived lineage, constituting one of the primary plastids-bearing hosts, and provides plastids for the secondary or tertiary endosymbiosis^1^. This phylum comprise two subphyla with seven classes^2^: the subphylum Cyanidiophytina composed of class Cyanidiophyceae, the other subphylum Rhodophytina composed of classes Florideophyceae, Bangiophyceae, Compsopogonophyceae, Stylonematophyceae, Rhodellophyceae and Porphyridiophyceae. Rhodophyta are primarily marine in distribution, with less than 3% of the over 6500 species occurring in truly freshwater habitats^3, 4^. Freshwater red algae are usually important constituents of stream floras, either in terms of abundance or distribution from local scale to biomes. Members of freshwater Rhodophyta cover six classes (Bangiophyceae, Compsopogonophyceae, Florideophyceae, Porphyridiophyceae, Stylonematophyceae and Cyanidiophyceae) of total seven in the Rhodophyta except for the Rhodellophyceae^5^, with some lineages exclusively inhabit in freshwater such as the Batrachospermales and Thoreales in Florideophyceae. Red algae in freshwater habitats tend to be macroscopic and benthic, meanwhile exhibiting a smaller size range than the marine members^6^. Their morphology varied from unicellular to multicellular forms including tufts, crusts and filaments. Reproductive types of freshwater red algae exhibit a diversity including cell division, monosporangia formation, carpogonium and spermatangium, and the last occurred during sexual reproduction and life history alternation^5^. The probable origin of these freshwater forms among the red algae, whether they were dwellers of inland waters or immigrants from the sea, is still in debate. Skuja considered the Cryptonemiales, Nemastomales, and especially of the Ceramiales representing higher types in freshwater Rhodophyta probably are migrants from the sea while the other members are primary types and have arisen from original inhabitants of inland waters based on the morphological and ecological features^7^. However, there are few molecular evidence for the origin of freshwater Rhodophyta up to now.

Chloroplast genomes of the red algae have high gene capacity and compact structure^8, 9^, and the mitochondria genomes of Florideophyceae are highly conserved despite a wide variety of morphological divergence^10^. Both chloroplast and mitochondrial genomes have been applied in the phylogenomic analysis of Rhodophyta^10–12^, and all have proved to capable to elucidate phylogenetic relationships at deep and terminal branches, thus reflecting evolutionary history between Rhodophyta and other eukaryotic lineages. The organelle genomes provide insights not only on phylogenetic relationship but also other features (genome size and structure, GC content, gene loss and genome synteny) involving in the genome evolution^13^. Other studies concerning organelle genomes of algae have also proved that organelle genomes can provide new insights into organelle function and evolution^14^. Furthermore the non-recombinant nature of organelles makes them good tools for inferring ancient phylogenetic relationships^15^. Considering the evolutionary status of Rhodophyta in plant kingdom, the chloroplast and mitochondrial genome evolution in Rhodophyta is of great importance to understand the organelle evolution in general.

Here we sequenced the organelle genomes of three members in freshwater Rhodophyta and analyzed their unique architecture and gene content features. Additionally, the available genome sequence of freshwater Rhodophyta in the GenBank were combined together to construct a phylogeny of Rhodophyta based on genome-wide information. We proposed a primary inference on the origin and evolutionary history of freshwater Rhodophyta, which needs to be testified with more sequence data and wider sampling.

## Results

### Organization and gene content of organelle genomes for freshwater Rhodophyta representatives

**Figure 1:**
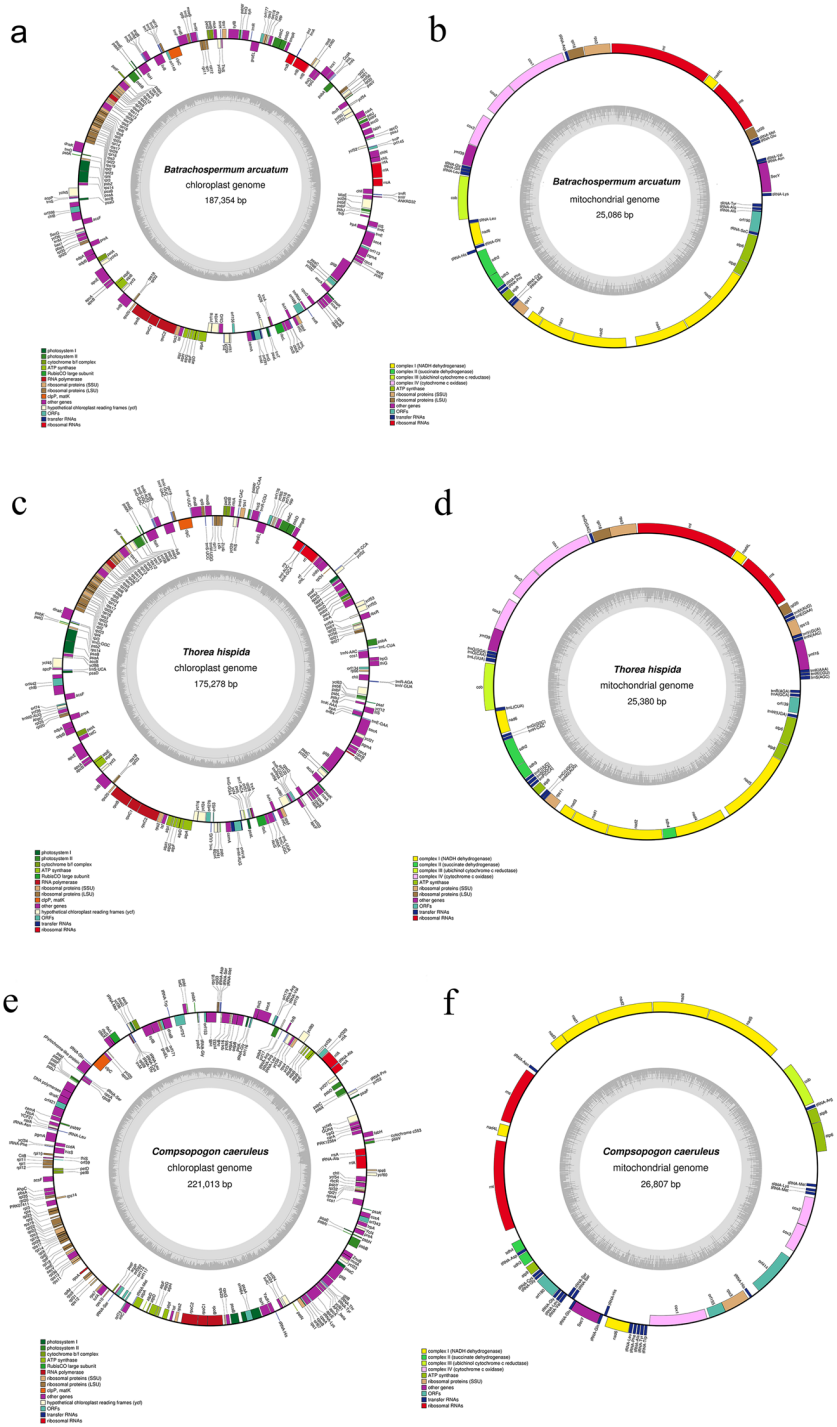
Organelle genome maps of freshwater Rhodophyta representatives. The genes inside and outside of the circles are transcribed in the clockwise and counterclockwise directions, respectively. Genes belonging to different functional groups are shown in different colors. (**a**) Chloroplast genome map of *Batrachospermum arcuatum*. (**b**) Mitochondrial genome map of *Batrachospermum arcuatum*. (**c**) Chloroplast genome map of *Thorea hispida*. (**d**) Mitochondrial genome map of *Thorea hispida*. (**e**) Chloroplast genome map of *Compsopogon caeruleus*. (**f**) Mitochondrial genome map of *Compsopogon caeruleus*.

The resulting assembly generated complete circular genomes for organelles of all the three freshwater species. *Batrachospermum arcuatum* Kylin generated a contig of 187,354 bp with 449 coverage for chloroplast and 25,086 bp with 757 coverage for mitochondria (Fig. 1a for chloroplast, b for mitochondria). *Thorea hispida* Desvaux generated a contig of 175,278 bp with 700 coverage for chloroplast and 25,380 bp with 300 coverage for the mitochondria (Fig. 1c for chloroplast, d for mitochondria). *Compsopogon caeruleus* Montagne generated a contig of 221,013 bp with 880 coverage for chloroplast, 26,807 bp with 1130 coverage for mitochondria (Fig. 1e for chloroplast, f for mitochondria). GC contents of the chloroplast are 29.9%, 28.3% and 26.0%, and of mitochondria are 29.7%, 28.2% and 27.4%, respectively for *Batrachospermum arcuatum*, *Thorea hispida* and *Compsopogon caeruleus*.

The *Batrachospermum arcuatum* chloroplast genome encodes 221 genes including 185 protein coding genes, 31 tRNAs, 6 rRNAs, 1 ncRNA (rnpB) and 1 tmRNA (Suppl. Table S1), and the mitochondria genome encodes 43 genes including 23 protein coding genes, 20 tRNAs and 2 rRNAs (Suppl. Table S2). The *Thorea hispida* chloroplast genome encodes 228 genes including 193 protein coding genes, 30 tRNAS, 3 rRNAs, 1 ncRNA (rnpB) and 1 tmRNA (Suppl. Table S3), and the mitochondria genome encodes 49 genes including 25 protein coding genes, 22 tRNAs and 2 rRNAs (Suppl. Table S4). The *Compsopogon caeruleus* chloroplast genome encodes 229 genes including 211 protein coding genes, 20 tRNAs and 5 rRNAs (Suppl. Table S5), and the mitochondria genome encodes 45 genes including 21 protein coding genes, 22 tRNAs and 2 rRNAs (Suppl. Table S6).

Two introns were discovered in genes *chl*B and *trn*M for the chloroplast genome of *Batrachospermum arcuatum* and *Thorea hispida*. 15 introns were discovered in the chloroplast genome of *Compsopogon caeruleus* distributed in genes of *odp*B, *clp*C, phytochrome-like protein, *pgm*A, *Ahp*C, *rp1*2, *rps*3, *inf*C, *atp*F, *atp*I, *rpo*C1, *psa*A, *psa*C, *psb*B and *dna*K. No introns were found in the mitochondrial genomes of the three species.

### Loss of tRNAs in the freshwater Rhodophyta members

Necessary tRNAs except *trn*N and *trn*P were found in the chloroplast genome of *Batrachospermum arcuatum*, whereas for the mitochondria genome absence of *trn*R, *trn*I, *trn*T, *trn*W and *trn*Y was observed. For *Thorea hispida*, all necessary tRNAs were found in the chloroplast genomes whereas absence of *trn*I, *trn*T and *trn*Y occurred in the mitochondria genome. In *Compsopogon caeruleus*, necessary tRNAs except *trn*E and *trn*I were found in the chloroplast genome and in the mitochondria genome the *trn*I and *trn*T have been lost.

### Synteny analysis and structure comparison

**Figure 2:**
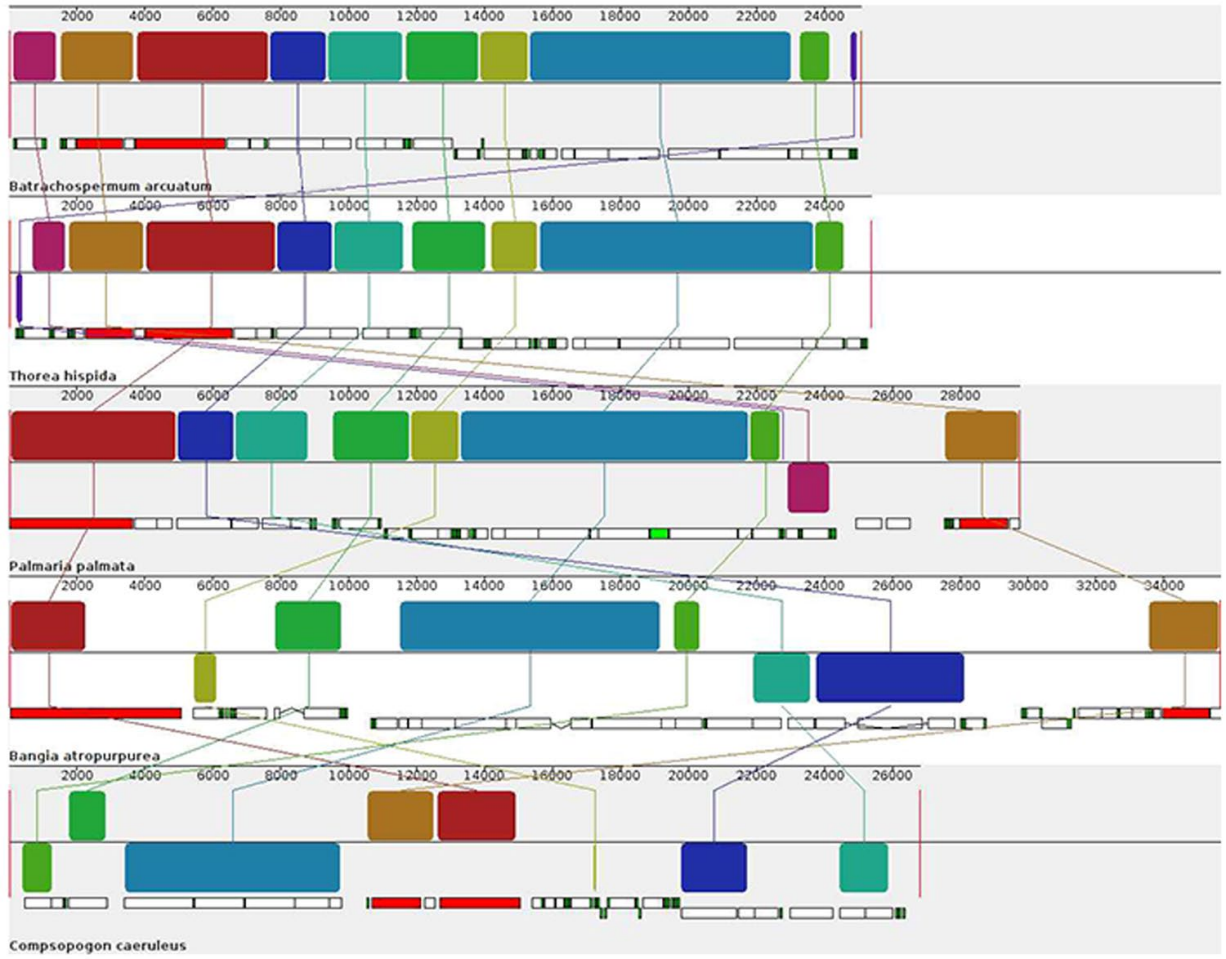
Synteny alignment of mitochondrial genomes between freshwater representatives. Each colored bar represents a locally collinear block and is assigned a unique color. White, red and green boxes represent annotated CDS (protein-coding sequence), rRNAs and tRNAs in the genomes respectively. Sequences each colored bar covered are homologous among the aligned genomes. The colored lines connect similar colored blocks and indicate which regions in each genome are homologous.

**Figure 3:**
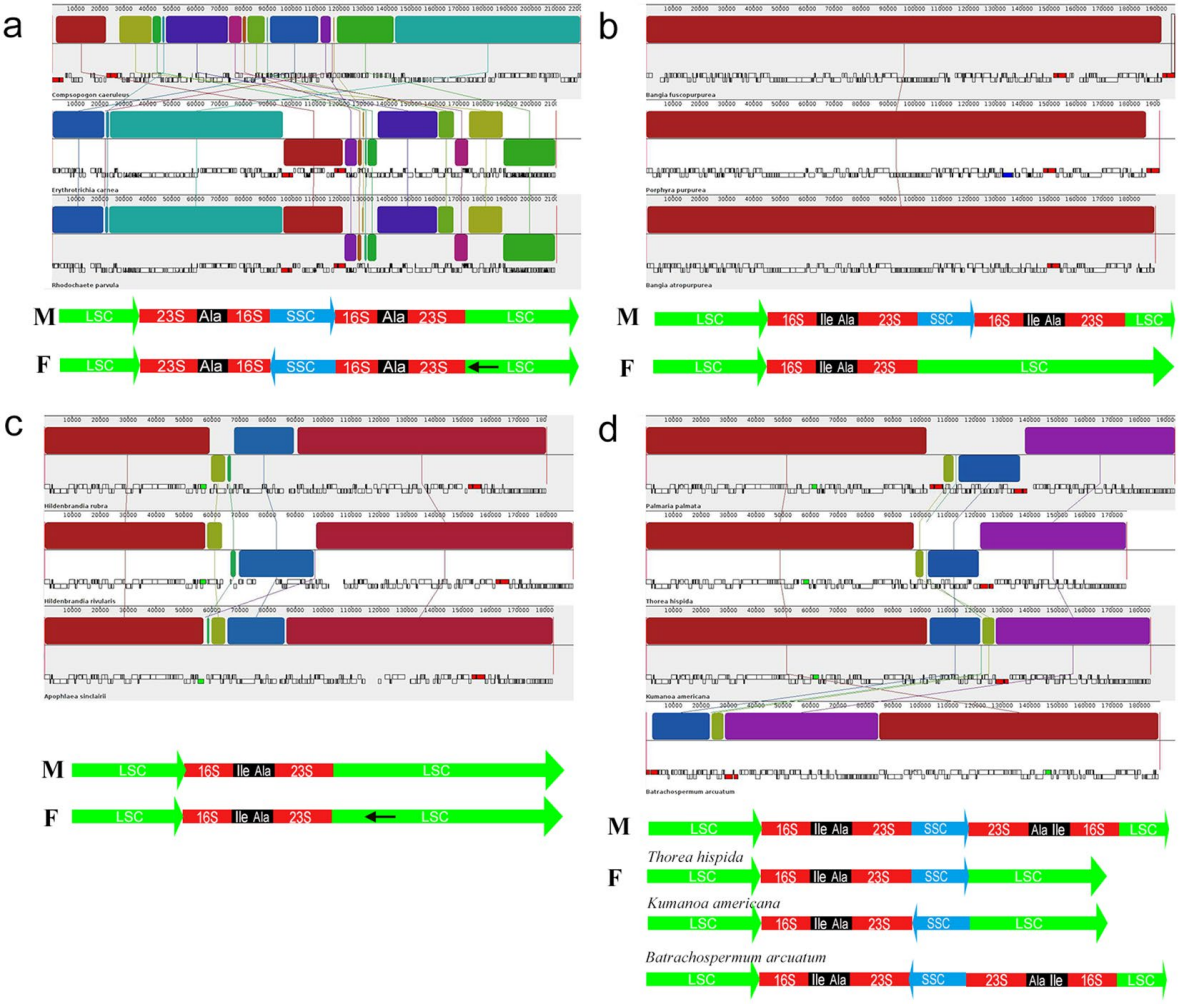
Synteny alignment of chloroplast genomes between freshwater and marine representatives.﻿ (**a**) Synteny alignment of chloroplast genomes between freshwater and marine Compsopogonophyceae. (**b**) Synteny alignment of chloroplast genomes between freshwater and marine Bangiophyceae. (**c**) Synteny alignment of chloroplast genomes between freshwater and marine Hildenbrandiophyceae. (**d**) Synteny alignment of chloroplast genomes between freshwater and marine Florideophyceae. Note: Lines with “M” and “F” mean types of chloroplast genome architecture for each Rhodophyta lineage. M represent marine species and F represent freshwater species; LSC and SSC represent genomic regions coding for large single copy and small single copy; 23 S and 16 S represent genes coding for 23 S rRNA and 16 S rRNA; Ala and Ile represent genes coding for tRNA-Ala and tRNA-Ile; Black arrows represent partial inverse of the LSC region. Lines connecting each colored bar with crossing “X” pattern represent genomic rearrangements or collinear structure annotated at different starting point.

The chloroplasts of the freshwater representatives (*Compsopogon caeruleus, Bangia atropurpurea, Hildenbrandia rivularis, Batrachospermum arcuatum, Kumanoa americana*) were poorly collinear, whereas the collinear alignment structure was observed for the mitochondrial genomes, particularly in the florideophyceae members (Crossing “X” pattern observed in lines connecting each colored bars was due to different annotation staring point) (Fig. 2). The mitochondrial genomes of Bangiophyceae and Compsopogonophyceae exhibited less collinear structure with that of the Florideophyceae. On the other hand, it was observed that freshwater and marine representatives from each Rhodophyta lineages shared high synteny when comparing their chloroplast genomes (Fig. 3). For the Compsopogonophyceae chloroplast, the freshwater and marine representatives all own 2 rRNA operons, which were identical in sequence while opposite in direction. A *trn*A was distributed in the region between 16 S rRNA and 23 S rRNA. The two marine members, *Erythrotrichia carnea* and *Rhodochaete parvula* share highly conservative structure, and the freshwater member *Compsopogon caeruleus* owned inverse SSC structure and partial inverse LSC rearrangement compared with the marine members (Fig. 3a). For the Bangiophyceae lineage, chloroplast genomes between freshwater and marine members had little structural rearrangements except for the loss of one rRNA operon in the freshwater member *Bangia atropurpurea* and the two rRNA operons in the marine members were arranged directly. Two tRNAs including *trn*I and *trn*A were distributed in the region of 16 S rRNA and 23 S rRNA (Fig. 3b). For the Hildenbrandiales in Florideophyceae, both freshwater and marine representatives own only one rRNA operon. Two rearrangements occurred between the marine members *Hildenbrandia rubra* and *Apophlaea sinclairii* and two larger blocks are rearranged between *H. rubra* and *H. rivularis*. One *trn*A was distributed in the region of 16 S rRNA and 23 S rRNA (Fig. 3c). For the Nemaliophycidae in Florideophceae, the marine representative owned two inverse rRNA operons and the freshwater members owned one or two rRNA operons. The freshwater member *Thorea hispida* shared highly collinear structure with the marine member *Palmaria palmate* except the loss of one rRNA operon. Chloroplast of *Kumanoa americana* owned one rRNA operon and was collinear with *Thorea hispida* except for the inverse arrangement of the SSC region. The *Batrachospermum arcuatum* were collinear with *Kumanoa americana* but gain one more rRNA operon and two rRNA operons were inverse arranged. Two tRNAs including *trn*I and *trn*A were distributed in the region between 16 S rRNA and 23 S rRNA (Fig. 3d).

### Phylogenetic relationship of freshwater Rhodophyta

**Figure 4:**
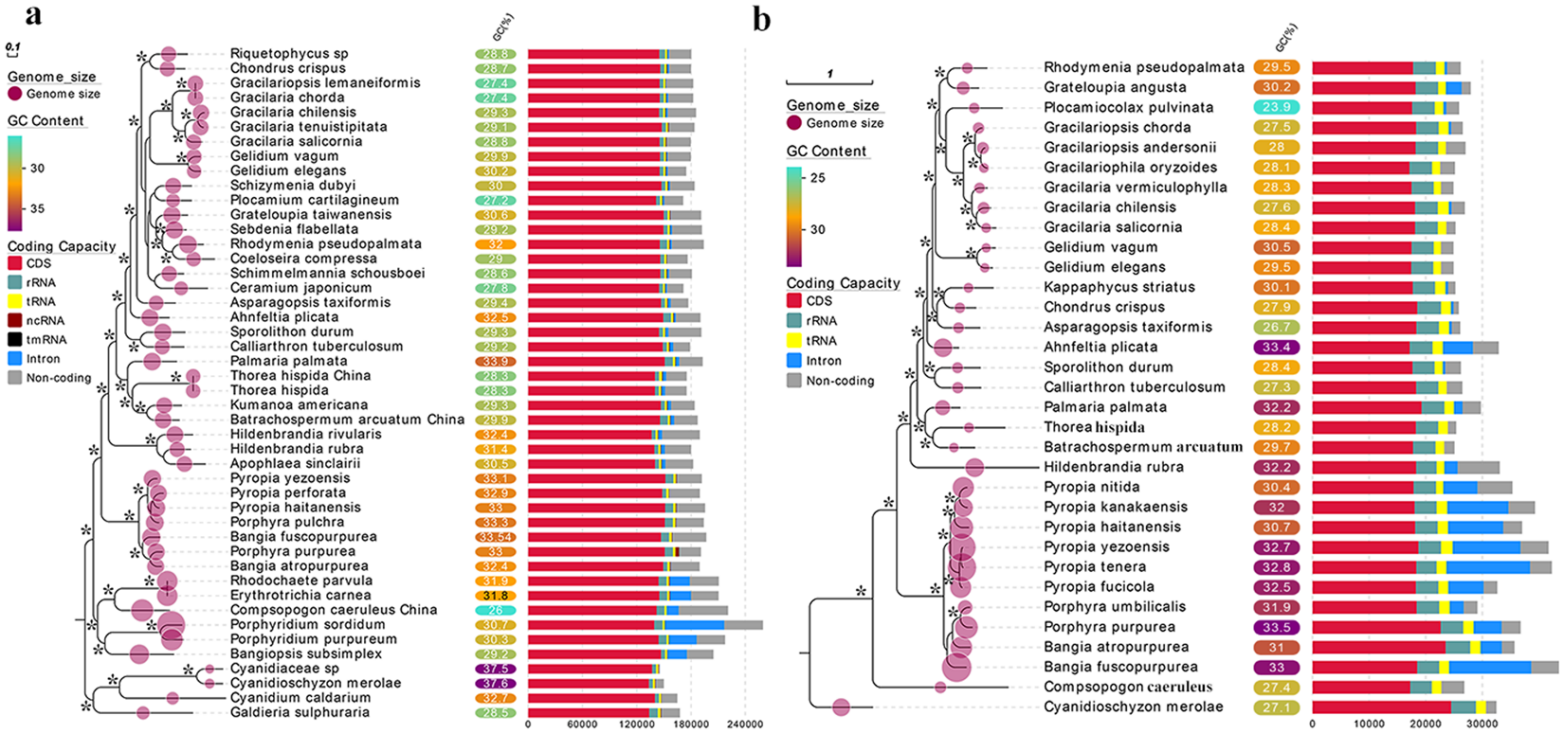
Bayesian phylogenetic trees based on organelle genomes, with each genomic features labeled on the tree. (**a**) Bayesian phylogenetic tree based on chloroplast genome. (**b**) Bayesian phylogenetic tree based on mitochondrial genome.﻿ Note: Asterisks on the nodes represent supporting values of Bayesian inference for 100% and Maximum Likelihood bootstrap more than 80%.

Both methods including Bayesian inference and maximum likelihood used for phylogenetic tree construction produced similar topologies, and only the Bayesian trees were showed with the Bayesian posterior probabilities and ML bootstrap supporting values labeled on the nodes (Fig. 4). Phylogeny based on chloroplast genome (Fig. 4a) revealed the freshwater members of each Rhodophyta lineage were distributed among the tree with robust supporting values. With the Cyanidiophyceae as the outgroup, the Porphyridiophyceae clustering together with the Compsopogonophyceae formed the basal branch, and the freshwater representative *Compsopogon caeruleus* was basal to the marine members *Erythrotrichia carnea* and *Rhodochaete parvula*. The second main branch contains classes of Bangiophyceae and Florideophyceae with the Bangiophyceae at the basal position. The freshwater representative in Bangiophyceae lineage, *Bangia atropurpurea* formed sister group with the marine member *Porphyra purpurea*. In the Hildenbrandiophycidae of Florideophyceae class, the freshwater representative *Hildenbrandia rivularis* was basal to the marine *Hildenbrandia rubra* and *Apophlaea sinclairii*, together formed the basal branch of Florideophyceae lineage. The typical freshwater members, Batrachospermales and Thoreales belonging to the Nemaliophycidae clustered together with the marine member *Palmaria palmate*. The Nemaliophycidae branch was basal to the other subclasses in Florideophyceae. As revealed by the genome size labeled on the branch, the Cyanidiophyceae lineage owned smallest chloroplast genome size, whereas the Porphyridiophyceae lineage owned the largest genome size. The genome size was decreasingly gradually from Porphyridiophyceae, Compsopogonophyceae, Bangiophyceae to Florideophyceae. In the Compsopogonophyceae lineage, the freshwater member *Compsopogon caeruleus* was slightly larger in chloroplast genome size than the marine members. In Bangiophyceae, freshwater representative owned smaller genome size than the marine representatives. In the Hildenbrandiophycidae, the freshwater *H. rivularis* are slightly larger than the marine member *Hildenbrandia rubra* and *Apophlaea sinclairii* in chloroplast genome size. In the Nemaliophycidae, the freshwater members *Thorea hispida*, *Batrachospermum arcuatum* and *Kumanoa americana* were slightly smaller than the marine member *Palmaria palmata* in chloroplast genome size. According to the results of the Spearman’s rank correlation tests, divergence of chloroplast genome size was principally caused by the variance of noncoding regions (*r* = 0.81, *p* = 0.000) and intron regions (*r* = 0.53, *p* = 0.000), whereas the length of protein-coding regions showed no significant difference in each Rhodophyta lineage. In Cyanidiophyceae, GC content of genus *Galdieria* was lower than other lineages. The GC content of Porphyridiophyceae, marine Compsopogonophyceae and Bangiophyceae were higher than the Florideophyceae. Totally, in all Rhodophyta lineages, freshwater representatives owns lower GC content than the marine representatives except for Hildenbrandiophycidae in Florideophyceae. Notably, the freshwater Compsopogonophyceae, *Compsopogon caeruleus* owned the lowest GC content in all the available Rhodophyta taxa.

Phylogeny based on mitochondria genome produced similar relationship with the chloroplast revealed (Fig. 4b). With no Porphyridiophyceae lineage organelle genome information, the *Compsopogon caeruleus* was at the basal position. The Bangiophyceae and Florideophyceae formed two main classes with full supporting values. The freshwater *Bangia atropurpurea* was distributed between the marine members *Bangia fuscopurpurea* and genus *Porphyra*. In Florideophyceae lineage, the freshwater *Thorea hispida* and *Batrachospermum arcuatum* formed a branch with full supporting values. This branch again clustered together with *Palmaria palmata*. Mitochondrial genome size of Florideophyceae was significantly smaller than that of Bangiophyceae according to the Mann-Whitney U test (*z* = −4.268, *p* = 0.000), and the genome size of Bangiophyceae were larger than that of Compsopogonophyceae and Cyanidiophyceae. The typical freshwater representatives, *Batrachospermum arcuatum* and *Thorea hispida* were smaller in mitochondrial genome size than its sister taxa, the marine *Palmaria palmata*. The GC content of the three freshwater representatives were lower than the marine members of each lineage.

### Divergence time estimation

**Figure 5:**
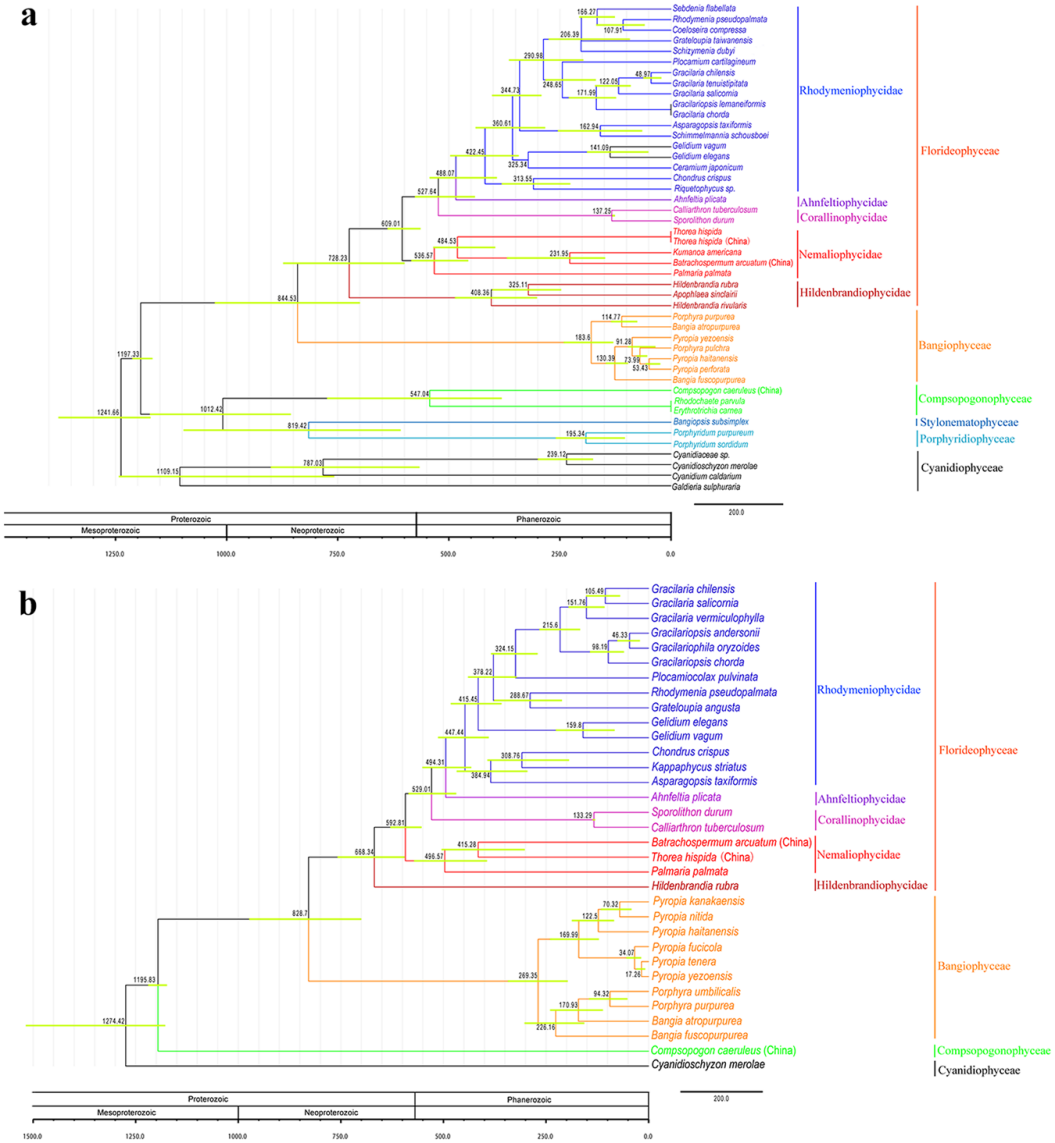
Divergence time estimation phylogram based on organelle genome sequence. Numbers at the nodes represent the mean estimated divergence time with the corresponding 95% highest posterior density (HPD) of the nodes indicated by horizontal bars. The geological timescale is given in million years ago (MYA). (**a**) Divergence time estimation tree based on the chloroplast genome sequence. (**b**) Divergence time estimation tree based on the mitochondrial genome sequence.

Divergence time estimation based on chloroplast genome (Fig. 5a) showed that the Porphyridiophyceae, Stylonematophyceae and Compsopogonophyceae diverged first, following the divergence of Bangiophyceae and Florideophyceae. In the Florideophyceae, the Hildenbrandiophycidae diverged first and the Nemaliophycidae followed. The freshwater member *Compsopogon caeruleus* diverged with marine members in class Compsopogonophyceae at 547 MYA (95% HPD: 384–777 MYA). The class Bangiophyceae diverged at approximate 844 MYA (95% HPD: 704–1030 MYA), and freshwater *Bangia atropurpurea* diverged later (approximately 114 MYA) than the marine *Bangia fuscopurpurea* (approximately 130 MYA). In Hildenbrandiophycidae of the class Florideophyceae, the freshwater *H. rivularis* diverged earlier at 408 MYA (95% HPD: 305–489 MYA) and marine *H. rubra* diverged at 325 MYA (95% HPD: 251–406 MYA). The typical freshwater representative *Thorea hispida* diverged from Batrachospermales at approximately 484 MYA (95% HPD: 399–538 MYA) and *Batrachospermum arcuatum* diverged at 231 MYA (95% HPD: 152–373 MYA).

As illustrated by the divergence time estimation based on mitochondrial genome (Fig. 5b), the divergence time of lineages Bangiophyceae and Florideophyceae was approximately similar to the result revealed by chloroplast genome (828 MYA vs 844 MYA for Bangiophyceae). However, due to the absent information of Porphyridiophyceae and Stylonematophyceae mitochondrial genomes, the class Compsopogonophyceae was at the basal position, and divergence time estimation of *Compsopogon caeruleus* was evidently earlier (1195 MYA) than the result of chloroplast genome (547 MYA). The divergence time of freshwater Bangiophyceae taxa *Bangia atropurpurea* (170 MYA) was earlier than the result of chloroplast (114 MYA). The divergence between marine *Palmaria palmata* and freshwater *Thorea hispida* and *Batrachospermum arcuatum* was 496 MYA, slightly later than the result of chloroplast (536 MYA).

### Gene loss between the freshwater and marine Rhodophyta

**Figure 6:**
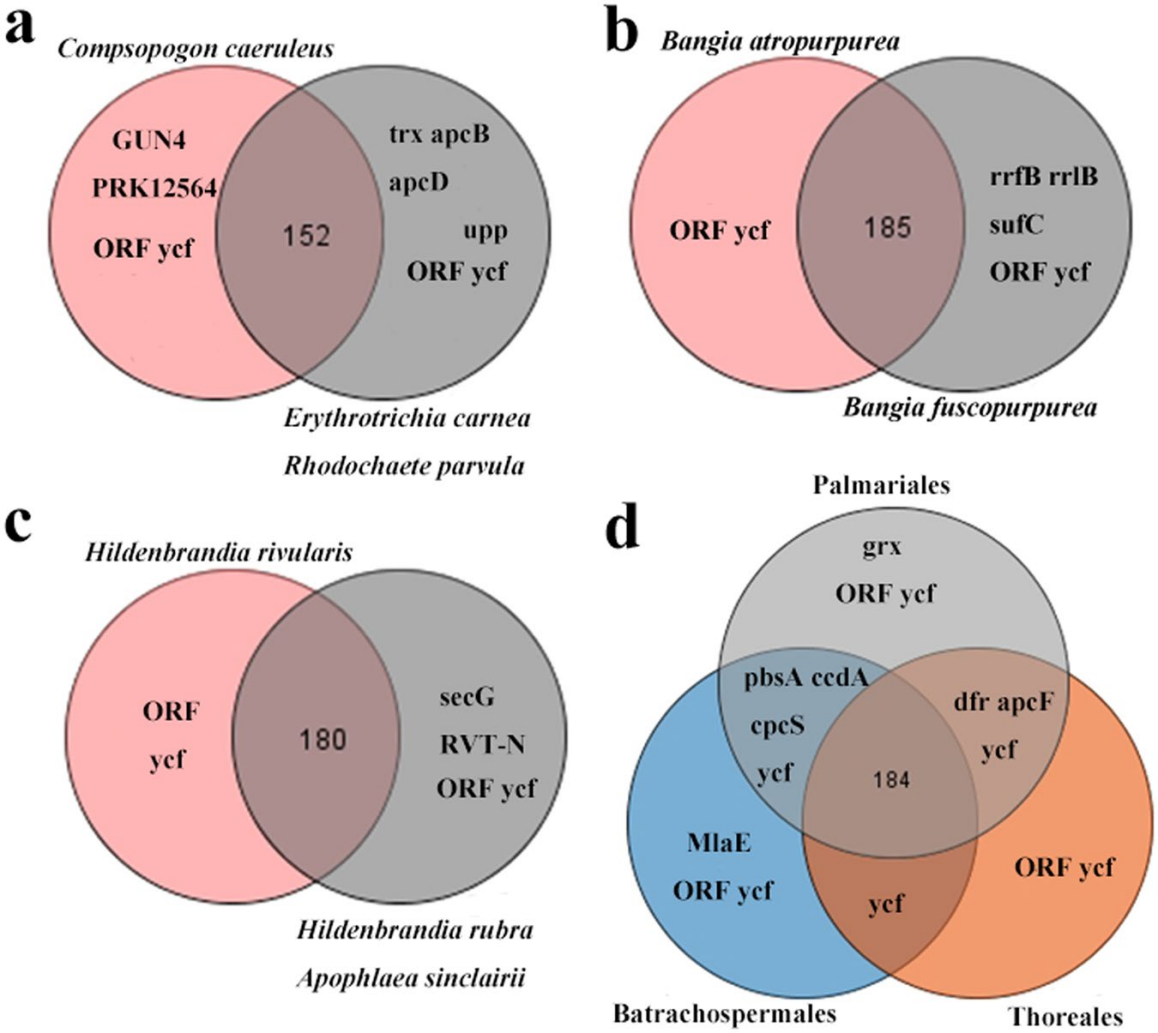
Venn diagram showing shared and unique gene content of each Rhodophyta class. (**a**) Venn diagram of gene content for the class Compsopogonophyceae. (**b**) Venn diagram of gene content for the class Bangiophyceae. (**c**) Venn diagram of gene content for the class Hildenbrandiophyceae. (**d**) Venn diagram of gene content for the class Florideophyceae.﻿ Note: Circles in gray represent marine members and circles in other colors represent freshwater members in each Rhodophyta lineage.

Unique chloroplast gene content of freshwater and marine representatives for each Rhodophyta lineage were illustrated in Fig. 6. Generally, ORF (open reading frame) and ycf (unknown reading frames) constituted most of the unique genes. Common genes loss, *trx* (thioredoxins) and *grx* (glutaredoxins) were noticed between the freshwater and marine members of Compsopogonophyceae and Florideophyceae (Fig. 6a,d). In Compsopogonophyceae, the freshwater representative *Compsopogon caeruleus* own unique protein-coding genes (GNU4, PRK12564) similar to cyanobacteria (Fig. 6a). For Bangiophyceae and Hildenbrandiophyceae, no special protein-coding genes were found in the freshwater members (Fig. 6b,c). Meanwhile, high conservation in mitochondrial gene contents were observed in Rhodophyta except that the *Compsopogon caeruleus* lost all the *rpl* genes (ribosomal protein large subunit).

### Substitution rates estimation of gene groups

**Figure 7:**
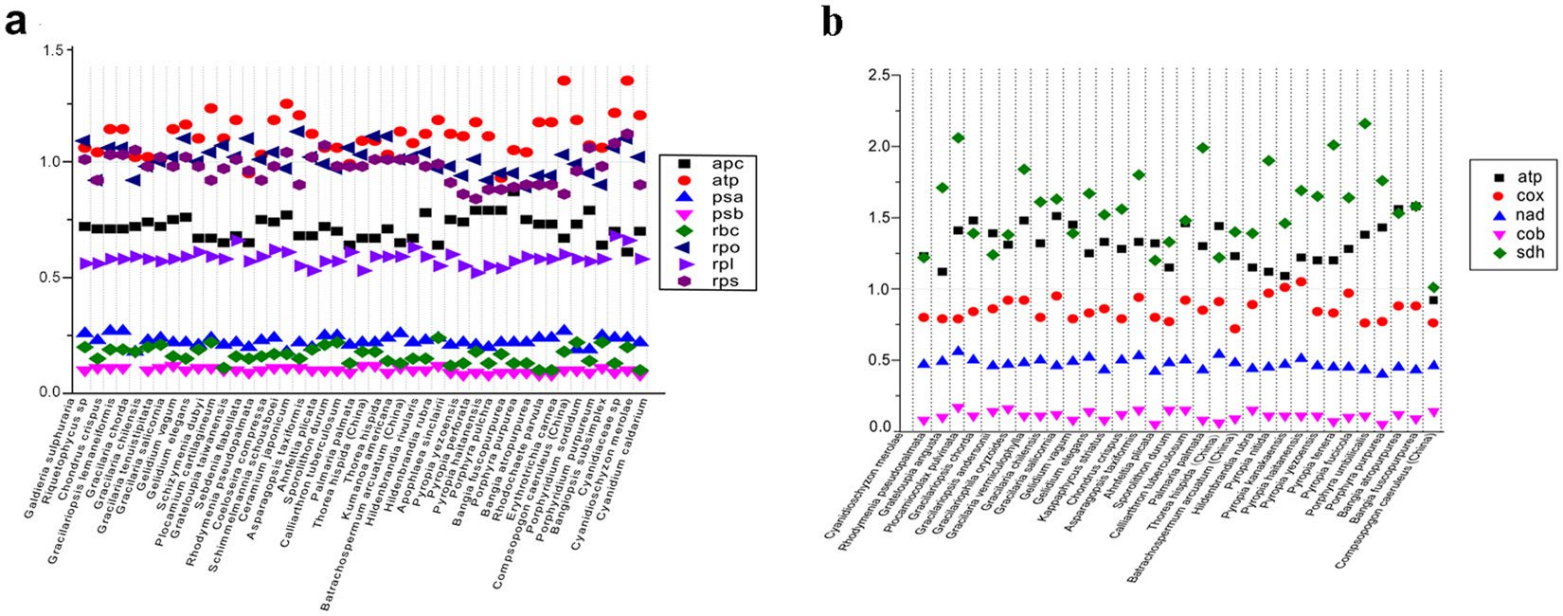
Substitution rates of organelle gene groups. (**a**) Substitution rates of chloroplast gene groups. (**b**) Substitution rates of mitochondrial gene groups.

Ratios of non-synonymous and synonymous substitutions of per gene group were calculated with the *Galdieria sulphuraria* and *Cyanidioschyzon merolae* as references (Suppl. Tables S7 and S8), as illustrated in Fig. 7. The ratios of non-synonymous and synonymous substitution for chloroplast gene groups including *psa*, *psb* and *rbc* were between 0 and 0.5, with *psa* the largest and *psb* the lowest. The ratios of *apc* and *rpl* were between 0.5 and 1.0 and the ratios of *atp*, *rps* and *rpo* were evidently higher with some species exceeding 1.0 (Fig. 7a). For mitochondrial gene groups, the ratio of *cob* was below 0.5, of the *nad* was around 0.5. Ratio of the cox was between 0.5 and 1.0 (except for genus *Pyropia*) and of the *atp* and *sdh* were higher than 1.0 (Fig. 7b). The difference of the nucleotide substitution rates among diverse gene groups were significant (*χ* ^*2*^ = 342.956, *p* = 0.000 for chloroplast gene groups; *χ* ^*2*^ = 147.493, *p* = 0.000 for mitochondria gene groups).

## Discussion

Of all the reported complete chloroplast genomes of multi-cellular freshwater Rhodophyta, *Compsopogon caeruleus* owned the largest genome size, and the chloroplast genome of *Batrachospermum arcuatum* and *Thorea hispida* were in the range of Florideophyceae^9, 12, 16, 17^. A possible history of expansion and subsequent contraction for the mitochondrial genome size has been discovered in green plant Cucurbitaceae^18^. Meanwhile, it has been proposed that all organelles undergo a genetic reduction, independently of their phylogenetic origin^19^. And the selection for small genomes contributes to the genome reduction^19^. Expansion and subsequent reduction of organelle genome size was observed in the red algae. The large-genome representatives Porphyridiophyceae, Stylonematophyceae and Compsopogonophyceae were more limited in species richness whereas the small-genome size representatives Bangiophyceae and Florideophyceae comprise greater than 98% of red algal diversity^4^. From the perspective of genomic size evolution, the freshwater members *Compsopogon caeruleus* and *Hildenbrandia rivularis* represented the primary forms in each class, and the *Bangia atropurpurea*, *Batrachospermum arcuatum* and *Thorea hispida* represented the evolved forms in each class.

Evidence have suggested that the pattern of spontaneous mutations is biased towards AT nucleotides in eukaryotes as well as Prokaryotes, thus decreasing the GC content^20, 21^. Šmarda *et al*. also argued the reduction process of genomic GC-content was probably ongoing and evolutionarily young^22^. The genomic GC content was decreasing with the evolution of Rhodophyta lineages, which was consistent with the general trend. However, the GC content of *Compsopogon caeruleus* was unexpectedly low contrasting its ancestral origin. *Compsopogon caeruleus* propagate by asexual reproduction and lack genetic recombination. Its low genetic variation has been found in previous record^23^. It is widely accepted that there exists positive correlation between genetic recombination and GC content^24^. Thus we speculated that the notably low GC content of *Compsopogon caeruleus* was caused by lack of genetic recombination. Considering the high GC content of *Hildenbrandia rivularis* comparing with its marine relatives, the freshwater *Hildenbrandia rivularis* represented the ancestral status. Whereas the contrary situation was observed in freshwater *Bangia atropurpurea*, *Thorea hispida* and *Batrachoseprmum arcuatum*, revealing their derived evolutionary stage.

Most of the early-diverged classes in Rhodophyta contained two copies of the ribosomal DNA operon, whereas single rDNA was found in most florideophycean species^12^. However, two inverted rDNAs were detected in the freshwater florideophycean taxa *Batrachospermum arcuatum*, whearas its sister genera *Kumanoa americana* and *Thorea hispida* owned single rDNA operon. It suggested that the loss and acquisition of rDNA in Rhodophyta were random and the structure of rDNA operon may be different even at closely-related taxa.

The mitochondria genome size of Bangiophyceae was significantly larger than that of the Florideophyceae and the GC content was higher than those of the Florideophyceae, which was consistent with previous report^10^. Unexpectedly, the mitochondrial genome of *Compsopogon caeruleus* exhibited an evidently small size compared with other ancient derived Rhodophyta lineages. The mitochondria genomes of freshwater Florideophycean representatives *Thorea hispida* and *Batrachospermum arcuatum* were highly conservative with its marine relative *Palmaria palmata* whereas extensive gene rearrangements occurred when comparing Compsopogonophycean, Bangiophyceae and Florideophyceae representatives. The similar phenomenon was also found in the marine Rhodophyta^10^. Additionally, introns were found in the marine Rhodophyta mitochondria genes, such as the *cox*1 and *trn*I^10^. Whereas no introns were found in all the freshwater representatives in this study, suggesting the independent evolution of freshwater and marine groups since their derivation.

Phylogenomic analysis in this study covered the freshwater and marine members of classes Compsopogonophyceae, Bangiophyceae and Florideophyceae, with the typical freshwater representatives Batrachospermales and Thoreales included. The class Compsopogonophyceae diverged at an earlier time followed by the sister group of Bangiophyceae and Florideophyceae, which was consistent with phylogenetic relationship in previous study^12^. Origin of freshwater Rhodophyta were lineage-specific based on phylogenomic data in this study, with the freshwater representatives *Compsopogon caeruleus* and *Hildenbrandia rivularis* diverging earlier than the marine relatives and probably originated and evolved independently at the inland water. Whereas the freshwater taxa *Bangia atropurpurea* in class Bangiophyceae and the typical freshwater representatives Thoreales and Batrachospermales were derived from the marine relatives. The results based on molecular data were partially consistent (on the origin of freshwater primary Rhodophyta like *Compsopogon caeruleus* and *Hildenbrandia rivularis*) with that proposed by Skuja based on morphological and ecological evidence^7^, whereas we expanded the scope of marine migrants origination to include freshwater Bangiales, Thoreales and Batrachospermales. Molecular-based inference on freshwater Rhodophyta origination and evolution were poorly studied before. The primary proposal in this study provided molecular evidence for further investigation of freshwater Rhodophyta.

It has proved difficult to resolve class-level relationships among red algae using multigene data^2, 25, 26^. Yoon *et al*. used seven plastid-encoded proteins and two rDNA sequences to construct phylogenetic tree of Rhodophyta and highlighted the inherent difficulties in resolving the intra-lineage relationship of the red algae except for the position of the Cyanidiales and the monophyly of Bangiales and Florideophyceae^2^. Phylogenetic trees generated in this study were robustly supported at most nodes, thus resolving the phylogenetic relationship at both the inter- and intra-class level for Rhodophyta and demonstrating the organelle genomes powerful tools for resolving the red algal phylogeny.

Based on the chloroplast phylogenomic analysis in this study, the Porphyridiophyceae, Stylonematophyceae and Compsopogonophyceae originated at 1012 MYA and the Bangiophyceae and Florideophyceae diverged 844 MYA (chloroplast genome based) vs 828 MYA (mitochondria genome based), which were similar to previous results based on multi-gene analysis. Yoon postulated the Porphyridiophyceae, Compsopogonophyceae, Stylonematophyceae, Rhodellophyceae and Bangiophyceae radiated around 1200 MYA, followed the divergence of Florideophyceae at around 800 MYA^2, 27^. Yang *et al*. suggested the Florideophyceae diverged 943 MYA beginning with the split of the Hildenbrandiophycidae followed by the divergence of Nemaliophycidae, Corallinophycidae, Ahnfeltiophycidae and Rhodymeniophycidae^28^. Phylogenomic data inferred the Compsopogonophyceae originated at Mesoproterozoic era and the Florideophyceae originated at Neoproterozoic era, which was consistent with the multi-gene evidence. The freshwater representatives *Compsopogon caeruleus*, *Thorea hispida* and *Batrachospermum arcuatum* diverged at 547 MYA, 484 MYA and 231 MYA respectively, all at the Phanerozoic era. Previous proposal based on morphological characterization highlighted the earlier divergence of Thoreales compared with other Nemaliophycidae lineages^29^. Genomic evidence was consistent with the morphological features in revealing the earlier divergence of Thoreales among Nemaliophycidae. Divergence of freshwater members occurred together with the radiation of the rhodymeniophycidaen algae^28^.

Thioredoxins (*Trx*) and glutaredoxins (*grx*) constituted families of thiol oxidoreductases and involved in disulphide/dithiol interchange. Marine algae produced large amounts of sulfated extracellular polysaccharides and sulfation accounted for a much greater proportion of the total assimilated sulfur^30^. Whereas polysaccharides of freshwater Rhodophyta characterized for *Batrachospermum* and *Compsopogon* species contained no ester sulfate^31^. *Trx* and *grx* genes have proved absent in the chloroplast genomes of *Batrachospermum arcuatum* and *Compsopogon caeruleus* in this study. Thus it was inferred that the *trx* and *grx* genes involved in the polysaccharides synthesis of Rhodophyta. No protein-coding unique genes were found in mitochondrial genomes of freshwater and marine Rhodophyta suggest the high conservation of gene content between these two groups.

Ka/Ks is significantly elevated for ribosomal protein (*rpl* and *rps*), RNA polymerase genes (*rpo*) and ATPase genes (*atp*) in the angiosperm plastid genomes^32^. Similarly, the three gene groups were proved increased in Rhodophyta chloroplast genomes, illustrating positive selection or relaxed selection in the Rhodophyta evolution. It was found that all ribosomal protein genes and *sdh* (succinate dehydrogenase) genes have been lost from the mitochondrial genome many times during angiosperm evolution^33^. In Rhodophyta, substitution rates are highly elevated for the *sdh* genes and all the *rpl* genes (ribosomal protein large subunits) have been lost in the mitochondrial genome of *Compsopogon caeruleus*. Given the ancient derivation of Rhodophyta, we inferred the features of high substitution rates for the chloroplast gene groups of ribosomal protein, RNA polymerase and ATPase, in combination with mitochondrial ribosomal protein genes and *sdh* genes, were shared by eukaryotic lineages produced through the primary and second endosymbiotic events. Variant substitution rates of each gene group can serve as candidate DNA barcoding at different taxonomic levels in the Rhodophyta systematics.

## Methods

### Taxon sampling

Algal specimens were collected from Nanlaoquan, Jinci Park and Niangziguan, Shanxi Province respectively (Table 1). Fresh thalli were first washed to eliminate the epiphytes and then desiccated in silica gel to be stored at −20 °C.

**Table 1:** Specimen information used in this study.

Species	Order and Class	Locality, latitude and longitude, collection date	GenBank accession numbers for chloroplast	GenBank accession numbers for mitochondria
*Batrachospermum arcuatum*	Batrachoserpmales, Florideophyceae	Nanlao Spring, Taiyuan, Shanxi province, China (37.71 N, 112.43E) March 2006.	KY033529	KY083064
*Thorea hispida*	Thoreales, Florideophyceae	Niangziguan, Pingding, Shanxi province, China (37.78 N, 113.52E) March 2009.	KY083065	KY083066
*Compsopogon caeruleus*	Compsopogonales, Compsopogonophyceae	Weizeguan Spring, Shanxi province, China (37.96 N; 113.88E) August 2013.	KY083067	KY083068

### Genome sequencing, Assembly and Annotation

Total DNA of algal specimens were sequenced using the Illumina Hiseq 2500 technology with 350 bp insertion fragments. The Illumina-generated reads were assembled with SPAdes 3.8.2^34^. Contigs generated were blast against other Rhodophyta chloroplast and mitochondrial genomes. Matched contigs with similar coverage were screened out and extended by a baiting and iteration method using the Price software^35^. The resulting contigs were loaded as reference sequences in Bowtie 2.1.0^36^ and the matched reads were used for another assembly under SPAdes 3.8.2. After iterative extension and assembly, the final circular structures were generated.

Annotation were conducted with Unipro UGENE for initial open reading frame (ORF) finding and blastp for annotation of protein-coding sequences (CDS)^37^. Large and small subunits of ribosomal RNA (rRNA) were identified using BLASTn with published red algal rRNAs as queries, and transfer RNAs (tRNA) and tmRNA were identified using using the tRNAscan-SE Search Server (http://lowelab.ucsc.edu/tRNAscan-SE/). The ribonuclease P gene (*rnp*B) was detected using Bcheck online web server^38^. Newly generated organelle genomes were deposited in GenBank.

### Similarity and structure comparison of organelle genomes

Chloroplast and mitochondrial genomes of *Batrachospermum arcuatum*, *Thorea hispida* and *Compsopogon caeruleus* were aligned with other Rhodophyta organelle genomes (Suppl. Table S9) using Mauve ver. 2.3.1 under the progressive mode^39^. The alignment result file was used to extract syntenic alignments by customized Perl scripts.

### Phylogenomic analysis and divergence time estimation

Sequence alignment at the genome-scale was used to construct phylogenetic trees. Optimal evolutionary models of the dataset were determined using Modeltest version 3.7^40^. Maximum likelihood^41^ under the RaxML with 1000 bootstrap replicates^42^ were used to construct the trees. Bayesian inference^43^ was run for 1,100,000 generations with sampling every 100 generations under the temperature of 0.2. After discarding the first 25% of trees as burn-in, posterior probabilities were calculated under the MrBayes^44^. Organelle genome length, GC content and features (length of CDS, rRNA, tRNA, ncRNA, tmRNA, intron, non-coding region) of each taxa in the phylogenetic tree were labeled on the tree using online tools EvolView (http://www.evolgenius.info/evolview.html). SPSS ver. 16.0 (SPSS, Inc., Chicago, IL, USA) was used for statistical analyses. Appropriate statistical analysis were selected after testing for the normal distribution of the datasets. Primary components leading to overall genome size variation were analyzed using Spearman’s rank correlation analysis. The significance of difference in mitochondrial genome size was analyzed using Mann-Whitney U test. The alpha level for all the two-tailed tests was 0.01. Divergence time estimation were inferred based on a Bayesian tree using BEAST2^45^. The analysis was performed under GTR nucleotide substitution model (determined by Modeltest in the phylogenetic analysis) with a Gamma distribution for four rate categories. Uncorrelated lognormal relaxed clock model was employed to account for the uncertainty in the divergence time estimation^46^. Calibration nodes were constrained based on three fossil records including the 1174–1222 MYA Bangiomorph multicellular red algae^47^, corallinalean algae from the Doushantuo Foamation at 635–551 MYA^48, 49^, and 130–136 MYA for the Sporolithales split^50^. Posterior distributions of parameters were approximated after 50,000,000 generations of MCMC runs, sampling every 5,000 generations with a 25% burn-in, and the divergence tree was visualized using FigTree^51^. The priors on the age of the nodes were set as a normal distribution as the normal approach has been proved more appropriate for red algae divergence time calculation^28^.

### Gene loss and substitution rates

The coding content of organelle genome were compared between the freshwater Rhodophyta and marine members for each lineage using TBtools Venn analysis (www.omicshare.com/tools). Substitution rates of the genes were analyzed using DnaSP ver. 5 with the outgroup in the phylogenetic trees as reference^52^. Genes were grouped according to their function. Eight gene groups for chloroplast genome including allophycocyanin (*apc*), ATP synthase genes (*atp*), photosystem I genes (*psa*), photosystem II genes (*psb*), rubisco gene (*rbc*), ribosomal protein (*rpo*) and RNA polymerase (*rpl* and *rps*) were concatenated to estimate nonsynonymous substitutions (Ka), synonymous substitutions (Ks), and their ratios (Ka/Ks). And the gene groups selected for the mitochondrial genome were ATP synthase genes (*atp*), cytochrome b (*cob*), cytochrome c oxidase (*cox*), NADH dehydrogenase (*nad*), and cytochrome c oxidoreductase (*sdh*). The relative ratios were illustrated in scatter plot using OriginPro 2016 (OriginLab Corporation USA). The significance of difference in substitution rates for diverse gene groups was tested using Kruskal-Wallis H test.

## Electronic supplementary material

Supplementary information
