# Supplementary material for: Origin and evolutionary history of freshwater Rhodophyta: further insights based on phylogenomic evidence
Source: Sci Rep. 2017 Jun 7;7:2934. doi: 10.1038/s41598-017-03235-5 (PMC5462760; doi:10.1038/s41598-017-03235-5)
Supplement: Supplementary file 1 — Supplementary information [file 41598_2017_3235_MOESM1_ESM.doc]

**Origin and evolutionary history of freshwater Rhodophyta: further insights based on phylogenomic evidence**

Fangru Nan^1^, Jia Feng^1^, Junping Lv^1^, Qi Liu^1^, Kunpeng Fang^1^, Chaoyan Gong^1^ and Shulian Xie^1^*

^1^School of Life Science, Shanxi University, Taiyuan (030006), China

*Corresponding author: E-mail: [xiesl@sxu.edu.cn](mailto:xiesl@sxu.edu.cn)

Supplementary Table S1 Chloroplast genome content of *Batrachospermum arcuatum*

| Rubiso | rbcL | rbcS | rbcR |  |  |  |  |
| --- | --- | --- | --- | --- | --- | --- | --- |
| Photosystem I | psaA | psaB | psaC | psaD | psaE | psaJ | psaK |
|  | psaL |  |  |  |  |  |  |
| Photosystem II | psbA | psbB | psbC | psbD | psbE | psbF | psbH |
|  | psbJ | psbK | psbN | psbV | psbW | psbY | psbZ |
| ATP synthase | atpA | atpB | atpD | atpE | atpF | atpG | atpH |
|  | atpI |  |  |  |  |  |  |
| Cytochrome b/f | petA | petB | petD | petF | petJ |  |  |
| Ribonuclease | rne |  |  |  |  |  |  |
| RNA polymerase | rpoA | rpoB | rpoC1 | rpoC2 |  |  |  |
| Hypothetical reading  frames (ycf) | ycf52 | ycf53 | ycf55 | ycf54 | ycf56 | ycf60 | ycf16 |
|  | ycf29 | ycf45 | ycf36 | ycf43 | ycf3 | ycf16 | ycf24 |
|  | ycf39 | ycf41 | ycf4 | ycf22 | ycf61 | ycf26 |  |
| Other genes | ChlL | ChlN | apcD | fabH | accD | carA | CcdA |
|  | ccs1 | trpG | thiG | ompR | upp | groEL | syh |
|  | syfB | thiS | ntcA | moeB | dnaB | ClpC | ilvB |
|  | argB | ftsH | secY | dnaK | accB | accP | ChlB |
|  | acsF | SecG | basl | pbsA | preA | odpA | odpB |
|  | apcA | apcB | apcE | infB | tsf | CbiQ | CemA |
|  | trxA | abbX | ilvH | infC | cpcG | nb1A | cpeB |
|  | cpeA | ccsA | accA | gltB | rpcB | cpcA | pgmA |
|  | secA | trpA | tilS | ftrB | MlaE | ChlI | tufA |
| Ribosomal protein | rpl1 | rpl11 | rpl12 | rpl13 | rpl14 | rpl16 | rpl18 |
|  | rpl19 | rpl2 | rpl20 | rpl21 | rpl22 | rpl23 | rpl24 |
|  | rpl27 | rpl28 | rpl29 | rpl3 | rpl31 | rpl32 | rpl33 |
|  | rpl35 | rpl36 | rpl4 | rpl5 | rpl6 | rpl9 | rps1 |
|  | rps10 | rps11 | rps12 | rps13 | rps14 | rps16 | rps17 |
|  | rps18 | rps19 | rps2 | rps20 | rps3 | rps4 | rps5 |
|  | rps6 | rps7 | rps8 | rps9 |  |  |  |
| clpP, matK | clpC |  |  |  |  |  |  |
| ORF | orf145 | orf177 | orf149 | orf398 | orf156 | orf766 | orf391 |
|  | orf488 | orf121 | orf268 | orf133 |  |  |  |
| Ribosomal RNAs | rrn5S | rrn16S | rrn23S | rrn5S | rrn16S | rrn23S |  |
| Tranfer RNAs | trnA | trnC | trnD | trnE | trnF | trnG | trnH |
|  | trnI | trnK | trnL | trnM | trnQ | trnR | trnS |
|  | trnT | trnV | trnW | trnY |  |  |  |
| Other RNA | tmRNA | rnpB |  |  |  |  |  |

*Supplementary Table S2 Mitochondria genome content of Batrachospermum arcuatum*

| tRNAs | trnA | trnN | trnD | trnC | trnQ | trnE | trnG | trnG |
| --- | --- | --- | --- | --- | --- | --- | --- | --- |
|  | trnH | trnL | trnL | trnK | trnM | trnM | trnF | trnP |
|  | trnS | trnV |  |  |  |  |  |  |
| rRNAs | rnl | rns |  |  |  |  |  |  |
| Complex I (NADH dehydrogenase) | nad1 | nad2 | nad3 | nad4 | nad4L | nad5 | nad6 |  |
| Complex II (succinate dehydrogenase) | sdh2 | sdh3 |  |  |  |  |  |  |
| Complex III (ubiquinone cytochrome c reductase) | cob |  |  |  |  |  |  |  |
| Complex IV(cytochrome c oxidase) | cox1 | cox2 | cox3 |  |  |  |  |  |
| ATP synthase | atp6 | atp8 | atp9 |  |  |  |  |  |
| Ribosomal proteins | rpl16 | rpl20 | rps11 | rps3 |  |  |  |  |
| Conserved ORFs | ymf39 | orf190 |  |  |  |  |  |  |
| other gene | secY |  |  |  |  |  |  |  |

Supplementary Table S3 Chloroplast genome content of *Thorea hispida*

| Rubiso | rbcL | rbcS | rbcR |  |  |  |  |
| --- | --- | --- | --- | --- | --- | --- | --- |
| Photosystem I | psaA | psaB | psaC | psaD | psaE | psaF | psaI |
|  | psaJ | psaK | psaL |  |  |  |  |
| Photosystem II | psbA | psbB | psbC | psbD | psbE | psbF | psbH |
|  | psbI | psbJ | psbK | psbL | psbN | psbT | psbV |
|  | psbW | psbX | psbY | psbZ |  |  |  |
| ATP synthase | atpA | atpB | atpD | atpE | atpF | atpG | atpH |
|  | atpI |  |  |  |  |  |  |
| Cytochrome b/f | petA | petB | petD | petF | petG | petJ | petL |
| Ribonuclease | rne |  |  |  |  |  |  |
| RNA polymerase | rpoA | rpoB | rpoC1 | rpoC2 | rpoZ |  |  |
| Hypothetical reading  frames (ycf) | ycf12 | ycf16 | ycf19 | ycf20 | ycf21 | ycf23 | ycf24 |
|  | ycf29 | ycf3 | ycf36 | ycf38 | ycf39 | ycf4 | ycf41 |
|  | ycf45 | ycf52 | ycf53 | ycf54 | ycf55 | ycf56 | ycf63 |
|  | ycf65 | ycf80 | ycf86 |  |  |  |  |
| Other genes | accA | accB | accD | acsF | AhpC | apcA | cbbX |
|  | apcB | apcD | apcE | apcF | apcP | argB | carA |
|  | ccs1 | ccsA | cemA | chlB | chlI | chlL | chlN |
|  | cpcA | cpcB | cpcG | cpeA | cpeB | dnaB | dnaK |
|  | fabH | ftrB | ftsH | gltB | groEL | hisS | ilvB |
|  | ilvH | infB | infC | moeB | nblA | ntcA | odpA |
|  | odpB | ompR | pgmA | preA | secA | secY | tatC |
|  | thiG | thiS | tilS | trpA | trpG | trxA | tufA |
|  | tsf | upp |  |  |  |  |  |
| Ribosomal protein | rpl1 | Rpl11 | rpl12 | rpl13 | rpl14 | rpl16 | rpl18 |
|  | rpl19 | rpl2 | rpl20 | rpl21 | rpl22 | rpl23 | rpl24 |
|  | rpl27 | rpl28 | rpl29 | rpl3 | rpl31 | rpl32 | rpl33 |
|  | rpl34 | rpl35 | rpl4 | rpl5 | rpl6 | rpl9 | rps1 |
|  | rps10 | rps11 | rps12 | rps13 | rps14 | rps16 | rps17 |
|  | rps18 | rps19 | rps2 | rps20 | rps3 | rps4 | rps5 |
|  | rps6 | rps7 | rps8 | rps9 | rpl36 |  |  |
| clpP, matK | clpC |  |  |  |  |  |  |
| ORF | orf120 | orf134 | orf176 | orf276 | orf418 | orf442 | orf53 |
|  | orf57 | orf58 | orf64 | orf74 |  |  |  |
| Ribosomal RNAs | rrf | rrl | rrs |  |  |  |  |
| Tranfer RNAs | trnA | trnC | trnD | trnE | trnF | trnG | trnH |
|  | trnI | trnK | trnL | trnM | trnP | trnQ | trnR |
|  | trnS | trnT | trnV | trnW | trnY | trnN |  |
| Other RNA | tmRNA | rnpB |  |  |  |  |  |

Supplementary Table S4 Mitochondria genome content of *Thorea hispida*

| tRNAs | trnA | trnC | trnD | trnE | trnF | trnG | trnH | trnK |
| --- | --- | --- | --- | --- | --- | --- | --- | --- |
|  | trnL | trnL | trnM | trnN | trnP | trnQ | trnR | trnS |
|  | trnV | trnW |  |  |  |  |  |  |
| rRNAs | rnl | rns |  |  |  |  |  |  |
| Complex I (NADH dehydrogenase) | nad1 | nad2 | nad3 | nad4 | nad4L | nad5 | nad6 |  |
| Complex II (succinate dehydrogenase) | sdh2 | sdh3 | sdh4 |  |  |  |  |  |
| Complex III (ubiquinone cytochrome c reductase) | cob |  |  |  |  |  |  |  |
| Complex IV(cytochrome c oxidase) | cox1 | cox2 | cox3 |  |  |  |  |  |
| ATP synthase | atp6 | atp8 | atp9 |  |  |  |  |  |
| Ribosomal proteins | rpl16 | rpl20 | rps11 | rps12 | rps3 |  |  |  |
| Conserved ORFs | ycf16 | ycf39 | orf139 |  |  |  |  |  |

*Supplementary Table S5 Chloroplast genome content of* Compsopogon caeruleus

| Rubiso | rbcL | rbcS | rbcR |  |  |  |  |
| --- | --- | --- | --- | --- | --- | --- | --- |
| Photosystem I | psaA | psaB | psaC | psaD | psaE | psaF | psaI |
|  | psaK | psaL |  |  |  |  |  |
| Photosystem II | psbA | psbB | psbC | psbD | psbE | psbF | psbH |
|  | psbI | psbJ | psbK | psbL | psbN | psbV | psbW |
|  | psbX | psbY | psbZ |  |  |  |  |
| ATP synthase | atpA | atpB | atpD | atpE | atpF | atpG | atpH |
|  | atpI |  |  |  |  |  |  |
| Cytochrome b/f | petA | petB | petD | petF | petN |  |  |
| Ribonuclease | rne |  |  |  |  |  |  |
| RNA polymerase | rpoA | rpoB | rpoC1 | rpoC2 |  |  |  |
| Hypothetical reading  frames (ycf) | ycf17 | ycf19 | ycf20 | ycf21 | ycf23 | ycf24 | ycf27 |
|  | ycf3 | ycf33 | ycf34 | ycf35 | ycf36 | ycf39 | ycf4 |
|  | ycf46 | ycf52 | ycf54 | ycf60 | ycf61 | ycf65 | ycf80 |
| Other genes | acpP | acsF | AhpC | apcA | APC_beta | apcE | apcF |
|  | carA | cbbX | ccdA | ccs1 | ccsA | cemA | chlI |
|  | CitB | cpcA | cpcB | cpcG | cpeA | cpeB | cytochrome c553 |
|  | dnaB | dnaK | DNA polymerase | fabH | ftrB | ftsH | gltB |
|  | groEL | gtlB | GUN4 | hisS | ilvB | ilvH | infB |
|  | infC | mntA | nblA | NtcA | odpA | odpB | pbsA |
|  | pgmA | phytochrome-like protein | preA | PRK07411 | PRK12564 | secA | secY |
|  | sufC | syfB | tatC | thiG | thiS | trpA | trpG |
|  | trxM | tsf | tufA | YadH | ZnuB |  |  |
| Ribosomal protein | rpl1 | Rpl11 | rpl12 | rpl14 | rpl16 | rpl18 | rpl19 |
|  | rpl2 | rpl20 | rpl21 | rpl22 | rpl23 | rpl28 | rpl29 |
|  | rpl3 | rpl31 | rpl32 | rpl33 | rpl35 | rpl36 | rpl4 |
|  | rplE | rplF | rplI | rplM | rplX | rps1 | rps10 |
|  | rps11 | rps12 | rps13 | rps14 | rps16 | rps17 | rps18 |
|  | rps19 | rps2 | rps20 | rps3 | rps4 | rpsE | rps6 |
|  | rps7 | rps9 | rpmA |  |  |  |  |
| clpP, matK | clpC |  |  |  |  |  |  |
| ORF | orf116 | orf117 | orf153 | orf171 | orf174 | orf179 | orf262 |
|  | orf329 | orf343 | orf421 | orf464 | orf59 | orf67 | orf74 |
|  | orf757 |  |  |  |  |  |  |
| Ribosomal RNAs | rrfA | rrlA (2) | rrsA (2) |  |  |  |  |
| Tranfer RNAs | trnA | trnC | trnD | trnF | trnG | trnH | trnK |
|  | trnL | trnM | trnN | trnP | trnQ | trnR | trnS |
|  | trnT | trnV | trnW | trnY |  |  |  |

*Supplementary Table S6 Mitochondria genome content of* Compsopogon caeruleus

| tRNAs | trnA | trnN | trnD | trnC | trnQ | trnE | trnG | trnH |
| --- | --- | --- | --- | --- | --- | --- | --- | --- |
|  | trnL | trnK | trnM | trnF | trnP | trnS | trnV | trnW |
|  | trnY | trnR |  |  |  |  |  |  |
| rRNAs | rnl | rns |  |  |  |  |  |  |
| Complex I (NADH dehydrogenase) | nad1 | nad2 | nad3 | nad4 | nad4L | nad5 | nad6 |  |
| Complex II (succinate dehydrogenase) | sdh3 | sdh4 |  |  |  |  |  |  |
| Complex III (ubiquinone cytochrome c reductase) | cob |  |  |  |  |  |  |  |
| Complex IV(cytochrome c oxidase) | cox1 | cox2 | cox3 |  |  |  |  |  |
| ATP synthase | atp6 | atp8 | atp9 |  |  |  |  |  |
| Ribosomal proteins | rps3 |  |  |  |  |  |  |  |
| Conserved ORFs | orf155 | orf180 | orf414 |  |  |  |  |  |
| other gene | secY |  |  |  |  |  |  |  |

Supplementary Table S7 Substitution rates of gene groups in the chloroplast genome

| Taxon | apc | | atp | | psa | | psb | | rbc | | rpo | | rpl | | rps | |
| --- | --- | --- | --- | --- | --- | --- | --- | --- | --- | --- | --- | --- | --- | --- | --- | --- |
|  | Ks | Ka | Ks | Ka | Ks | Ka | Ks | Ka | Ks | Ka | Ks | Ka | Ks | Ka | Ks | Ka |
| *Ahnfeltia plicata* | 0.60 | 0.40 | 0.36 | 0.40 | 0.98 | 0.19 | 1.18 | 0.11 | 1.45 | 0.26 | 0.41 | 0.41 | 0.72 | 0.41 | 0.41 | 0.44 |
| *Apophlaea sinclairii* | 0.64 | 0.41 | 0.35 | 0.41 | 0.86 | 0.21 | 1.05 | 0.12 | 1.09 | 0.23 | 0.43 | 0.42 | 0.74 | 0.44 | 0.52 | 0.47 |
| *Asparagopsis taxiformis* | 0.55 | 0.38 | 0.32 | 0.39 | 0.81 | 0.18 | 1.02 | 0.11 | 1.55 | 0.24 | 0.39 | 0.40 | 0.74 | 0.39 | 0.42 | 0.43 |
| *Bangia atropurpurea* | 0.51 | 0.38 | 0.39 | 0.41 | 0.85 | 0.19 | 1.26 | 0.11 | 1.78 | 0.23 | 0.45 | 0.40 | 0.69 | 0.41 | 0.47 | 0.42 |
| *Bangia fuscopurpurea* | 0.50 | 0.39 | 0.18 | 0.17 | 0.88 | 0.20 | 1.25 | 0.11 | 1.62 | 0.24 | 0.23 | 0.27 | 0.86 | 0.47 | 0.47 | 0.42 |
| *Bangiopsis subsimplex* | 0.57 | 0.37 | 0.35 | 0.37 | 0.75 | 0.19 | 1.03 | 0.11 | 1.22 | 0.27 | 0.44 | 0.40 | 0.71 | 0.41 | 0.47 | 0.46 |
| *Batrachospermum arcuatum* | 0.55 | 0.36 | 0.37 | 0.42 | 0.70 | 0.18 | 1.07 | 0.12 | 1.67 | 0.23 | 0.39 | 0.40 | 0.64 | 0.40 | 0.44 | 0.44 |
| *Calliarthron tuberculosum* | 0.55 | 0.39 | 0.37 | 0.39 | 0.78 | 0.20 | 1.10 | 0.11 | 1.14 | 0.25 | 0.38 | 0.40 | 0.65 | 0.40 | 0.44 | 0.43 |
| *Ceramium japonicum* | 0.50 | 0.39 | 0.29 | 0.37 | 0.84 | 0.15 | 1.04 | 0.12 | 1.36 | 0.24 | 0.41 | 0.40 | 0.65 | 0.40 | 0.40 | 0.42 |
| *Chondrus crispus* | 0.55 | 0.39 | 0.36 | 0.38 | 0.81 | 0.18 | 1.01 | 0.11 | 1.54 | 0.24 | 0.41 | 0.38 | 0.72 | 0.40 | 0.46 | 0.42 |
| *Coeloseira compressa* | 0.53 | 0.40 | 0.38 | 0.39 | 0.79 | 0.18 | 1.11 | 0.11 | 1.37 | 0.21 | 0.41 | 0.42 | 0.70 | 0.41 | 0.49 | 0.45 |
| *Compsopogon caeruleus* | 0.54 | 0.36 | 0.29 | 0.40 | 0.73 | 0.20 | 1.06 | 0.11 | 1.30 | 0.23 | 0.38 | 0.40 | 0.66 | 0.39 | 0.49 | 0.42 |
| *Cyanidiaceae sp* | 0.66 | 0.46 | 0.42 | 0.50 | 0.84 | 0.20 | 1.30 | 0.12 | 1.97 | 0.25 | 0.46 | 0.49 | 0.81 | 0.55 | 0.56 | 0.60 |
| *Cyanidioschyzon merolae* | 0.73 | 0.45 | 0.39 | 0.53 | 0.86 | 0.21 | 1.28 | 0.13 | 1.41 | 0.25 | 0.44 | 0.49 | 0.83 | 0.55 | 0.54 | 0.60 |
| *Cyanidium caldarium* | 0.65 | 0.45 | 0.38 | 0.45 | 0.96 | 0.21 | 1.53 | 0.13 | 2.09 | 0.22 | 0.42 | 0.43 | 0.84 | 0.49 | 0.59 | 0.53 |
| *Erythrotrichia carnea* | 0.56 | 0.41 | 0.35 | 0.41 | 0.84 | 0.20 | 1.52 | 0.12 | 2.26 | 0.24 | 0.45 | 0.42 | 0.72 | 0.42 | 0.50 | 0.45 |
| *Gelidium elegans* | 0.54 | 0.41 | 0.33 | 0.39 | 0.81 | 0.18 | 1.05 | 0.10 | 1.61 | 0.24 | 0.37 | 0.41 | 0.70 | 0.41 | 0.43 | 0.44 |
| *Gelidium vagum* | 0.43 | 0.32 | 0.34 | 0.38 | 0.83 | 0.18 | 0.91 | 0.10 | 1.46 | 0.24 | 0.39 | 0.40 | 0.70 | 0.41 | 0.45 | 0.44 |
| *Gracilaria chilensis* | 0.59 | 0.43 | 0.39 | 0.40 | 0.75 | 0.18 | 1.07 | 0.11 | 1.66 | 0.23 | 0.44 | 0.41 | 0.72 | 0.43 | 0.44 | 0.46 |
| *Gracilaria chorda* | 0.57 | 0.40 | 0.35 | 0.40 | 0.70 | 0.19 | 1.03 | 0.11 | 1.24 | 0.23 | 0.37 | 0.39 | 0.69 | 0.40 | 0.42 | 0.44 |
| *Gracilaria salicornia* | 0.57 | 0.41 | 0.39 | 0.39 | 0.75 | 0.18 | 0.96 | 0.11 | 1.19 | 0.23 | 0.41 | 0.41 | 0.72 | 0.41 | 0.44 | 0.45 |
| *Gracilaria tenuistipitata* | 0.57 | 0.42 | 0.38 | 0.39 | 0.76 | 0.18 | 1.07 | 0.11 | 1.32 | 0.23 | 0.42 | 0.41 | 0.73 | 0.42 | 0.45 | 0.44 |
| *Gracilariopsis lemaneiformis* | 0.57 | 0.40 | 0.35 | 0.40 | 0.70 | 0.19 | 1.03 | 0.11 | 1.24 | 0.23 | 0.37 | 0.39 | 0.69 | 0.40 | 0.42 | 0.44 |
| *Grateloupia taiwanensis* | 0.62 | 0.40 | 0.37 | 0.40 | 0.89 | 0.18 | 1.13 | 0.11 | 1.98 | 0.23 | 0.39 | 0.42 | 0.70 | 0.41 | 0.45 | 0.43 |
| *Hildenbrandia rivularis* | 0.64 | 0.43 | 0.39 | 0.42 | 0.94 | 0.21 | 1.19 | 0.12 | 1.50 | 0.23 | 0.43 | 0.45 | 0.76 | 0.45 | 0.51 | 0.50 |
| *Hildenbrandia rubra* | 0.57 | 0.44 | 0.37 | 0.42 | 0.86 | 0.20 | 1.13 | 0.12 | 1.45 | 0.22 | 0.45 | 0.44 | 0.79 | 0.43 | 0.48 | 0.48 |
| *Kumanoa americana* | 0.54 | 0.38 | 0.36 | 0.37 | 0.77 | 0.19 | 1.21 | 0.11 | 1.55 | 0.23 | 0.40 | 0.40 | 0.68 | 0.40 | 0.41 | 0.42 |
| *Palmaria palmata* | 0.64 | 0.41 | 0.41 | 0.41 | 0.92 | 0.20 | 1.24 | 0.12 | 1.95 | 0.28 | 0.42 | 0.43 | 0.80 | 0.43 | 0.46 | 0.45 |
| *Plocamium cartilagineum* | 0.59 | 0.39 | 0.31 | 0.38 | 0.77 | 0.18 | 1.06 | 0.11 | 1.06 | 0.23 | 0.38 | 0.39 | 0.65 | 0.39 | 0.45 | 0.41 |
| *Porphyra pulchra* | 0.50 | 0.39 | 0.38 | 0.42 | 0.92 | 0.18 | 1.47 | 0.11 | 1.85 | 0.23 | 0.44 | 0.41 | 0.77 | 0.42 | 0.48 | 0.43 |
| *Porphyra purpurea* | 0.45 | 0.39 | 0.39 | 0.41 | 0.86 | 0.19 | 1.26 | 0.12 | 1.77 | 0.23 | 0.43 | 0.41 | 0.72 | 0.41 | 0.48 | 0.43 |
| *Porphyridium purpureum* | 0.54 | 0.42 | 0.38 | 0.40 | 0.97 | 0.18 | 1.25 | 0.11 | 1.74 | 0.24 | 0.44 | 0.42 | 0.76 | 0.43 | 0.44 | 0.47 |
| *Porphyridium sordidum* | 0.58 | 0.43 | 0.35 | 0.42 | 0.92 | 0.18 | 1.21 | 0.12 | 1.42 | 0.24 | 0.43 | 0.42 | 0.72 | 0.42 | 0.48 | 0.47 |
| *Pyropia haitanensis* | 0.49 | 0.38 | 0.35 | 0.41 | 0.88 | 0.19 | 1.29 | 0.11 | 1.41 | 0.22 | 0.44 | 0.40 | 0.76 | 0.42 | 0.48 | 0.42 |
| *Pyropia perforata* | 0.52 | 0.38 | 0.37 | 0.41 | 0.86 | 0.19 | 1.50 | 0.11 | 1.75 | 0.23 | 0.41 | 0.42 | 0.91 | 0.47 | 0.50 | 0.42 |
| *Pyropia yezoensis* | 0.50 | 0.37 | 0.37 | 0.42 | 0.85 | 0.18 | 1.27 | 0.12 | 2.15 | 0.22 | 0.44 | 0.42 | 0.75 | 0.42 | 0.48 | 0.42 |
| *Rhodochaete parvula* | 0.56 | 0.41 | 0.35 | 0.41 | 0.84 | 0.20 | 1.52 | 0.12 | 2.26 | 0.24 | 0.45 | 0.42 | 0.72 | 0.42 | 0.50 | 0.45 |
| *Rhodymenia pseudopalmata* | 0.63 | 0.41 | 0.42 | 0.40 | 0.98 | 0.19 | 1.21 | 0.11 | 1.50 | 0.23 | 0.40 | 0.44 | 0.74 | 0.42 | 0.47 | 0.45 |
| *Riquetophycus* sp*.* | 0.55 | 0.40 | 0.37 | 0.39 | 0.74 | 0.19 | 1.06 | 0.11 | 1.21 | 0.23 | 0.35 | 0.39 | 0.69 | 0.39 | 0.42 | 0.42 |
| *Schimmelmannia schousboei* | 0.54 | 0.40 | 0.33 | 0.39 | 0.76 | 0.18 | 1.04 | 0.11 | 1.31 | 0.23 | 0.37 | 0.39 | 0.65 | 0.40 | 0.42 | 0.41 |
| *Schizymenia dubyi* | 0.61 | 0.41 | 0.36 | 0.40 | 0.86 | 0.18 | 1.05 | 0.12 | 1.35 | 0.23 | 0.40 | 0.40 | 0.65 | 0.40 | 0.44 | 0.43 |
| *Sebdenia flabellata* | 0.58 | 0.39 | 0.33 | 0.39 | 0.84 | 0.19 | 1.14 | 0.12 | 1.33 | 0.21 | 0.40 | 0.41 | 0.61 | 0.40 | 0.42 | 0.43 |
| *Sporolithon durum* | 0.54 | 0.39 | 0.37 | 0.39 | 0.79 | 0.19 | 1.08 | 0.11 | 1.21 | 0.24 | 0.40 | 0.39 | 0.71 | 0.41 | 0.44 | 0.43 |
| *Thorea hispida (China)* | 0.57 | 0.39 | 0.37 | 0.40 | 0.88 | 0.19 | 1.01 | 0.12 | 1.25 | 0.22 | 0.36 | 0.40 | 0.70 | 0.41 | 0.42 | 0.42 |
| *Thorea hispida* | 0.57 | 0.39 | 0.37 | 0.40 | 0.88 | 0.20 | 1.01 | 0.12 | 1.25 | 0.22 | 0.36 | 0.40 | 0.70 | 0.42 | 0.42 | 0.42 |

Note: Ks denotes synonymous substitutions; Ka denotes nonsynonymous substitutions.

Supplementary Table S8 Substitution rates of gene groups in the mitochondrial genome

| Taxon | atp | | cob | | cox | | nad | | sdh | |
| --- | --- | --- | --- | --- | --- | --- | --- | --- | --- | --- |
|  | Ks | Ka | Ks | Ka | Ks | Ka | Ks | Ka | Ks | Ka |
| *Ahnfeltia plicata* | 0.33 | 0.44 | 3.73 | 0.17 | 0.46 | 0.37 | 1.09 | 0.45 | 0.36 | 0.43 |
| *Asparagopsis taxiformis* | 0.26 | 0.35 | 1.12 | 0.17 | 0.33 | 0.31 | 0.82 | 0.43 | 0.24 | 0.43 |
| *Bangia atropurpurea* | 0.30 | 0.46 | 1.60 | 0.20 | 0.41 | 0.36 | 0.98 | 0.44 | 0.31 | 0.47 |
| *Bangia fuscopurpurea* | 0.29 | 0.46 | 2.12 | 0.18 | 0.52 | 0.45 | 1.06 | 0.45 | 0.29 | 0.46 |
| *Batrachospermum arcuatum* | 0.34 | 0.41 | 2.23 | 0.20 | 0.46 | 0.33 | 0.93 | 0.44 | 0.31 | 0.43 |
| *Calliarthron tuberculosum* | 0.26 | 0.39 | 1.25 | 0.19 | 0.36 | 0.33 | 0.86 | 0.43 | 0.27 | 0.40 |
| *Chondrus crispus* | 0.32 | 0.41 | 1.57 | 0.18 | 0.43 | 0.34 | 0.89 | 0.44 | 0.26 | 0.40 |
| *Compsopogon caeruleus* | 0.42 | 0.39 | 1.72 | 0.24 | 0.47 | 0.36 | 0.94 | 0.43 | 0.93 | 0.94 |
| *Gelidium elegans* | 0.35 | 0.43 | 1.39 | 0.20 | 0.39 | 0.32 | 0.85 | 0.45 | 0.27 | 0.45 |
| *Gelidium vagum* | 0.30 | 0.44 | 2.30 | 0.19 | 0.43 | 0.34 | 0.91 | 0.45 | 0.36 | 0.50 |
| *Gracilaria chilensis* | 0.27 | 0.36 | 1.57 | 0.18 | 0.41 | 0.32 | 0.85 | 0.42 | 0.25 | 0.41 |
| *Gracilaria salicornia* | 0.26 | 0.40 | 1.52 | 0.18 | 0.35 | 0.34 | 0.94 | 0.44 | 0.27 | 0.44 |
| *Gracilaria vermiculophylla* | 0.25 | 0.37 | 1.66 | 0.18 | 0.38 | 0.35 | 0.88 | 0.43 | 0.22 | 0.40 |
| *Gracilariophila oryzoides* | 0.29 | 0.38 | 1.11 | 0.18 | 0.36 | 0.33 | 0.91 | 0.43 | 0.30 | 0.41 |
| *Gracilariopsis andersonii* | 0.28 | 0.39 | 1.30 | 0.18 | 0.39 | 0.33 | 0.94 | 0.43 | 0.34 | 0.43 |
| *Gracilariopsis chorda* | 0.26 | 0.38 | 1.64 | 0.18 | 0.41 | 0.34 | 0.84 | 0.42 | 0.31 | 0.43 |
| *Grateloupia angusta* | 0.33 | 0.37 | 1.84 | 0.18 | 0.41 | 0.33 | 0.91 | 0.44 | 0.26 | 0.45 |
| *Hildenbrandia rubra* | 0.37 | 0.42 | 1.68 | 0.26 | 0.44 | 0.39 | 1.04 | 0.46 | 0.36 | 0.51 |
| *Kappaphycus striatus* | 0.32 | 0.42 | 2.16 | 0.18 | 0.43 | 0.37 | 1.03 | 0.45 | 0.33 | 0.49 |
| *Palmaria palmata* | 0.33 | 0.43 | 2.30 | 0.18 | 0.38 | 0.32 | 1.02 | 0.44 | 0.23 | 0.46 |
| *Plocamiocolax pulvinata* | 0.24 | 0.34 | 1.16 | 0.19 | 0.41 | 0.32 | 0.76 | 0.43 | 0.21 | 0.43 |
| *Porphyra purpurea* | 0.32 | 0.46 | 3.73 | 0.19 | 0.48 | 0.37 | 1.13 | 0.45 | 0.30 | 0.52 |
| *Porphyra umbilicalis* | 0.33 | 0.46 | 1.79 | 0.19 | 0.46 | 0.35 | 1.01 | 0.44 | 0.22 | 0.47 |
| *Pyropia fucicola* | 0.31 | 0.40 | 1.93 | 0.19 | 0.59 | 0.57 | 1.00 | 0.45 | 0.28 | 0.46 |
| *Pyropia haitanensis* | 0.30 | 0.36 | 1.73 | 0.19 | 0.34 | 0.36 | 0.86 | 0.44 | 0.26 | 0.43 |
| *Pyropia kanakaensis* | 0.34 | 0.37 | 1.70 | 0.19 | 0.56 | 0.57 | 0.94 | 0.44 | 0.29 | 0.43 |
| *Pyropia nitida* | 0.32 | 0.35 | 1.69 | 0.19 | 0.78 | 0.75 | 0.97 | 0.44 | 0.24 | 0.46 |
| *Pyropia tenera* | 0.33 | 0.39 | 2.80 | 0.19 | 0.45 | 0.38 | 1.02 | 0.45 | 0.23 | 0.46 |
| *Pyropia yezoensis* | 0.32 | 0.39 | 1.79 | 0.19 | 0.44 | 0.37 | 0.99 | 0.45 | 0.27 | 0.44 |
| *Rhodymenia pseudopalmata* | 0.31 | 0.38 | 2.14 | 0.17 | 0.43 | 0.35 | 0.92 | 0.43 | 0.34 | 0.42 |
| *Sporolithon durum* | 0.32 | 0.37 | 1.30 | 0.19 | 0.42 | 0.32 | 0.91 | 0.43 | 0.35 | 0.46 |
| *Thorea hispida* | 0.30 | 0.43 | 3.73 | 0.23 | 0.39 | 0.36 | 0.86 | 0.46 | 0.40 | 0.48 |

Note: Ks denotes synonymous substitutions; Ka denotes nonsynonymous substitutions.

Supplementary Table S9 GenBank accession numbers of specimens used in this study

| Taxon | GenBank No. of chloroplast genome | GenBank No. of mitochondria genome | References |
| --- | --- | --- | --- |
| *Ahnfeltia plicata* | KX284715 | NC_026054 | 1,2 |
| *Apophlaea sinclairii* | KX284716 |  | 2 |
| *Asparagopsis taxiformis* | KX284717 | NC_026843 | 1,2 |
| *Bangia atropurpurea* | NC_030221 | NC_030222 | Unpublished |
| *Bangia fuscopurpurea* | KP714733 | NC_026905 | Unpublished |
| *Bangiopsis subsimplex* | KX284718 |  | 2 |
| *Calliarthron tuberculosum* | NC_021075 | NC_027061 | 3,unpublished |
| *Ceramium japonicum* | KX284719 |  | 2 |
| *Chondrus crispus* | NC_020795 | NC_001677 | 4,5 |
| *Coeloseira compressa* | NC_030338 |  | unpublished |
| *Cyanidiaceae sp.* | KJ569775 |  | unpublished |
| *Cyanidioschyzon merolae* | NC_004799 | NC_000887 | 6,7 |
| *Cyanidium caldarium* | NC_001840 |  | 8 |
| *Erythrotrichia carnea* | KX284721 |  | 2 |
| *Galdieria sulphuraria* | NC_024665 |  | 9 |
| *Gelidium elegans* | NC_029858 | NC_026053 | 1,10 |
| *Gelidium vagum* | NC_029859 | NC_023077 | 10,11 |
| *Gracilariophila oryzoides* |  | NC_014771 | 12 |
| *Gracilariopsis andersonii* |  | NC_014772 | 12 |
| *Gracilaria Salicornia* | NC_023785 | NC_023784 | 13,14 |
| *Gracilaria tenuistipitata var. liui* | NC_006137 |  | 13 |
| *Gracilaria chilensis* | NC_029860 | NC_026831 | 10,15 |
| *Gracilaria chorda* | KX284722 |  | 2 |
| *Gracilariopsis chorda* |  | NC_023251 | 16 |
| *Gracilaria vermiculophylla* |  | NC_027064 | unpublished |
| *Gracilariopsis lemaneiformis* | NC_029644 |  | 17 |
| *Grateloupia angusta* |  | NC_023094 | 18 |
| *Grateloupia taiwanensis* | NC_021618 |  | 19 |
| *Hildenbrandia rivularis* | KX284723 |  | 2 |
| *Hildenbrandia rubra* | KX284724 | NC_026055 | 1,2 |
| *Kappaphycus striatus* |  | NC_024265 | 20 |
| *Kumanoa Americana* | KX284725 |  | 2 |
| *Palmaria palmata* | KX284726 | NC_026056 | 1,2 |
| *Plocamium cartilagineum* | KX284727 |  | 2 |
| *Plocamiocolax pulvinata* |  | NC_014773 | 12 |
| *Porphyra purpurea* | NC_000925 | NC_002007 | 4,21 |
| *Porphyra pulchra* | NC_029861 |  | 10 |
| *Porphyra umbilicalis* |  | NC_018544 | 22 |
| *Porphyridium purpureum* | NC_023133 |  | 23 |
| *Porphyridium sordidum* | KX284720 |  | 2 |
| *Pyropia fucicola* |  | NC_024288 | 24 |
| *Pyropia kanakaensis* |  | NC_024289 | 24 |
| *Pyropia haitanensis* | NC_021189 | NC_017751 | 25,26 |
| *Pyropia nitida* |  | NC_027616 | 27 |
| *Pyropia perforate* | NC_024050 |  | 24 |
| *Pyropia tenera* |  | NC_021475 | 28 |
| *Pyropia yezoensis* | KC517072 | NC_017837 | 26,28 |
| *Rhodochaete parvula* | KX284728 |  | 2 |
| *Rhodymenia pseudopalmata* | KX284709 | NC_023252 | 2,11 |
| *Riquetophycus sp.* | KX284710 |  | 2 |
| *Schimmelmannia schousboei* | KX284711 |  | 2 |
| *Schizymenia dubyi* | KX284712 |  | 2 |
| *Sebdenia flabellate* | KX284713 |  | 2 |
| *Sporolithon durum* | NC_029857 | NC_023454 | 10,30 |
| *Thorea hispida* | KX284714 |  | 2 |

**Reference**

1. Yang, E. C. et al. Highly conserved mitochondrial genomes among multicellular red algae of the Florideophyceae. Genome Biol. Evol. **7**, 2394-2406 (2015).
2. Lee, J. M. et al. Parallel evolution of highly conserved plastid genome architecture in red seaweeds and seed plants. BMC Biol. **14**, 75 (2016).
3. Janouškovec, J. et al. Evolution of red algal plastid genomes: ancient architectures, introns, horizontal gene transfer, and taxonomic utility of plastid markers. PLoS One **8**, e59001 (2013).
4. Burger, G., Saintlouis, D., Gray, M. W. & Lang, B. F. Complete sequence of the mitochondrial DNA of the red alga *Porphyra purpurea*: cyanobacterial introns and shared ancestry of red and green algae. Plant Cell **11**, 1675-1694 (1999).
5. Collén, J. et al. Genome structure and metabolic features in the red seaweed *Chondrus crispus* shed light on evolution of the Archaeplastida. P. Natl. Acad. Sci. U S A. **110**, 5247-5252 (2013).
6. Ohta, N., Sato, N. & Kuroiwa, T. Structure and organization of the mitochondrial genome of the unicellular red alga *Cyanidioschyzon merolae* deduced from the complete nucleotide sequence. Nucleic Acids Res **26**, 5190-5198 (1998).
7. Ohta, N. et al. Complete sequence and analysis of the plastid genome of the unicellular red alga *Cyanidioschyzon merolae*. Dna Res. **10**, 67-77 (2003).
8. Glöckner, G., Rosenthal, A. & Valentin, K. The structure and gene repertoire of an ancient red algal plastid genome. J. Mol. Evol. **51**, 382-390 (2000).
9. Jain, K. et al. Extreme features of the *Galdieria sulphuraria* organellar genomes: A Consequence of Polyextremophily? Genome Biol. Evol. **7**, 367-380 (2015).
10. Lee, J. M. et al. Reconstructing the complex evolutionary history of mobile plasmids in red algal genomes. SCI Rep-UK. **6**, 23744 (2016).
11. Yang, E. C. et al. Complete mitochondrial genome of the agarophyte red alga *Gelidium vagum* (Gelidiales). Dna Res. **25**, 267-268 (2014).
12. Hancock, L., Goff, L. & Lane, C. Red Algae lose key mitochondrial genes in response to becoming parasitic. Genome Biol. Evol. **2**, 897-910 (2010).
13. Hagopian, J. C., Reis, M., Kitajima, J. P., Bhattacharya, D. & de Oliveira, M. C. Comparative analysis of the complete plastid genome sequence of the red alga *Gracilaria tenuistipitata var. liui* provides insights into the evolution of rhodoplasts and their relationship to other plastids. J. Mol. Evol. **59**, 464-477 (2004).
14. Campbell, M. A., Presting, G., Bennett, M. S. & Sherwood, A. R. Highly conserved organellar genomes in the Gracilariales as inferred using new data from the Hawaiian invasive alga *Gracilaria salicornia* (Rhodophyta). Phycologia **53**, 109-116 (2014).
15. Lee, J. M., Boo, S. M., Mansilla, A. & Yoon, H. S. Unique repeat and plasmid sequences in the mitochondrial genome of *Gracilaria chilensis* (Gracilariales, Rhodophyta). Phycologia **54**, 20-23 (2015).
16. Yang, E. C., Kim, K. M., Su, Y. K. & Yoon, H. S. Complete mitochondrial genome of agar-producing red alga *Gracilariopsis chorda* (Gracilariales). Mitochondr DNA **25**, 339-341 (2014).
17. Du, Q., Bi, G., Mao, Y. & Sui, Z. The complete chloroplast genome of *Gracilariopsis lemaneiformis* (Rhodophyta) gives new insight into the evolution of family Gracilariaceae. J. Phycol. **52**, 441-450 (2016).
18. Kim, S. Y., Yang, E. C., Boo, S. M. & Yoon, H. S. Complete mitochondrial genome of the marine red alga *Grateloupia angusta* (Halymeniales). Mitochondr DNA 25, 269-270 (2014).
19. Depriest, M. S., Bhattacharya, D. & Lópezbautista, J. M. The plastid genome of the red macroalga *Grateloupia taiwanensis* (Halymeniaceae). Plos One **8**, e68246 (2013).
20. Tablizo, F. A. & Lluisma, A. O. The mitochondrial genome of the red alga *Kappaphycus striatus* ("Green Sacol" variety): complete nucleotide sequence, genome structure and organization, and comparative analysis. Mar. Genom. **18**, 155-161 (2014).
21. Reith, M. & Munholland, J. Complete nucleotide sequence of the *Porphyra purpurea* chloroplast genome. Plant Mol. Biol. Rep. **13**, 333-335 (1995).
22. Smith, D. R., Hua, J., Lee, R. W. & Keeling, P. J. Relative rates of evolution among the three genetic compartments of the red alga *Porphyra* differ from those of green plants and do not correlate with genome architecture. Mol. Phylogenet. Evol. **65**, 339-344 (2012).
23. Tajima, N. et al. Analysis of the complete plastid genome of the unicellular red alga *Porphyridium purpureum*. J. Plant Res. **127**, 389-397 (2014).
24. Hughey, J. R. et al. Minimally destructive sampling of type specimens of *Pyropia* (Bangiales, Rhodophyta) recovers complete plastid and mitochondrial genomes. SCI Rep-UK **4**, 5113 (2014).
25. Mao, Y., Zhang, B., Kong, F. & Wang, L. The complete mitochondrial genome of *Pyropia haitanensis* Chang et Zheng. Mitochondr DNA. **23**, 344-346 (2012).
26. Wang, L. et al. Complete sequence and analysis of plastid genomes of two economically important red algae: *Pyropia haitanensis* and *Pyropia yezoensis*. PLoS One **8**, e65902 (2013).
27. Harden, L. K., Morales, K. M. & Hughey, J. R. Identification of a new marine algal species *Pyropia nitida* sp. nov. (Bangiales: Rhodophyta) from Monterey, California. Mitochondr DNA **27**, 3058-3062 (2016).
28. Mi, S. H., Kim, S. O., Ha, D. S., Lee, J. E. & Lee, S. R. Complete sequence and genetic features of the mitochondrial genome of *Pyropia tenera* (Rhodophyta). Plant Biotechnol Rep. **7**, 435-443 (2013).
29. Kong, F., Sun, P., Cao, M., Wang, L. & Mao, Y. Complete mitochondrial genome of *Pyropia yezoensis*: reasserting the revision of genus *Porphyra*. Mitochondr DNA. **25**, 335-336 (2014).
30. Kim, K. M., Yang, E. C., Kim, J. H., Nelson, W. A. & Yoon, H. S. Complete mitochondrial genome of a rhodolith, *Sporolithon durum* (Sporolithales, Rhodophyta). Mitochondr DNA. **26**, 155-156 (­­­2015).
